# Diagnostic accuracy of Xpert MTB/RIF for tuberculosis detection in different regions with different endemic burden: A systematic review and meta-analysis

**DOI:** 10.1371/journal.pone.0180725

**Published:** 2017-07-14

**Authors:** Shiying Li, Bin Liu, Mingli Peng, Min Chen, Wenwei Yin, Hui Tang, Yuxuan Luo, Peng Hu, Hong Ren

**Affiliations:** Key Laboratory of Molecular Biology for Infectious Diseases (Ministry of Education), Institute for Viral Hepatitis, Department of Infectious Diseases, The Second Affiliated Hospital, Chongqing Medical University, Chongqing, PR, China; McGill University, CANADA

## Abstract

**Purpose:**

To estimate the diagnostic accuracy of Xpert MTB/RIF, a systematic review and meta-analysis were carried out.

**Methods:**

Up to June 20, 2015, multiple databases were screened for relevant studies.

**Results:**

Accordingly, 106 studies included 52,410 samples were selected. Diagnostic accuracy of Xpert MTB/RIF for TB detection was validated against either culture or a composite reference standard (CRS). Additionally, selected studies were further subgrouped in four groups based on sample’s type, subject’s age, status of HIV co-infection and smear-positivity. The overall pooled sensitivity and specificity of Xpert MTB/RIF was 0.85 (95% confidence interval [CI] 0.82–0.88) and 0.98 (95% CI 0.96–0.98), respectively, compared to culture; while it was 0.59 (95% CI 0.44–0.72) and 0.99 (95% CI 0.97–1.00) compared to CRS. The overall sensitivity was lower in countries with high TB prevalence than countries with middle/low prevalence (0.84, 95% CI: 0.80–0.88 versus 0.89, 95% CI: 0.84–0.93). Furthermore, Xpert MTB/RIF has higher sensitivity in patients with positive smears (0.99, 95% CI 0.97–0.99), in patients with pulmonary TB samples (0.87, 95% CI 0.83–0.90), in adults (0.82, 95% CI 0.76–0.86) and in HIV-positive patients (0.81, 95% CI 0.73–0.87).

**Conclusions:**

Taken together, Xpert MTB/RIF is a quick and accurate diagnostic assay for TB which will significantly help the physicians to make their clinical decisions.

## Introduction

Tuberculosis (TB) is a serious global health problem and is one of the leading causes of death worldwide. In 2013, an estimated 9.0 million people were infected with TB disease and 1.5 million (approximately 17%) died from this disease. Importantly, these recent epidemiological estimates are higher than what were previously estimated. Notably, of the 1.5 million deceased cases, 360,000 recorded among people with HIV infection.[1]

Despite of its life-threating pathogenesis, TB is a curable disease when it is correctly diagnosed and effectively treated. However, rapid and accurate diagnosis of TB can be difficult due to the paucibacillary characteristics of the disease (especially for cases with smear-negative, co-infection with HIV and drug-resistance) and the challenge of sample collection from deep-seated tissues.[2,3] In fact, approximately 35% of all the worldwide TB infections are undiagnosed.[4] Furthermore, the ratio of patients with undiagnosed multi-drug resistant TB remains much staggering (~75%). [4] Less than 3% of patients who are diagnosed with TB infection are proved to have certain pattern of drug resistance.[5]

Solid and/or liquid culture is generally considered as the standard reference for TB diagnosis. However, limited laboratory facilities in resource-limited settings and prolonged culturing period restrict the utility of culture-based diagnosis in clinical practice.[6] Histology is widely applied to the diagnosis of TB where the technical expertise exists, but this is technically demanding, time-consuming and it lacks specificity.[7] In early 2011, a novel, automated, rapid, cartridge-based nucleic acid amplification test, named the Xpert® MTB/RIF assay (Cepheid, Sunnyvale, USA) was authorized by the World Health Organization (WHO) to be used for TB diagnosis.[8] Xpert® MTB/RIF can simultaneously test both TB and rifampicin resistance through examination of the DNA of *Mycobacterium* tuberculosis and detection of major mutations which confer rifampicin resistance.[9] This assay was first endorsed by WHO as an initial diagnostic test in patients with human immunodeficiency virus (HIV)-associated pulmonary TB or suspected pulmonary MDR-TB.[10] Xpert® MTB/RIF showed a substantial accuracy for detection of pulmonary TB in adults with 89% sensitivity and 99% specificity.[11] In late 2013, WHO expanded its recommendations to include the diagnosis of TB in some special subjects such as children and patients with certain forms of extrapulmonary TB.[1] A systematic review by Detjen et al. revealed that Xpert offers a better sensitivity (62%) and specificity (98%) for the diagnosis of pulmonary tuberculosis in children.[12] However, the information concerned the accuracy of Xpert MTB/RIF in different TB endemic areas is lacking.

In fact, the prevalence of TB is clearly varying among different regions. Based on global TB epidemiology in 2013, 56% of TB cases worldwide were in the Western Pacific Regions and South-East Asia while 25% of the cases were in the African Region, which also had the highest rates of cases and deaths relative to population. Notably, India and China alone had 24% and 11% of total cases, respectively.[1] Therefore, in this systemic review, we aimed to determine the diagnostic accuracy of Xpert MTB/RIF assay in different regions with different TB prevalence regardless of sample type, subject’s age, HIV co-infection or smear-positivity.

## Methods

Following the standard guidelines, we designed a protocol before commencing the study. [13,14]

### Literature search strategy

MEDLINE, EMBASE, the Cochrane Library, and Web of Knowledge were used to retrieve published work without language or date restrictions. The last search was done on June 20, 2015. The keywords used for searching were: ‘‘Xpert”, ‘‘Gene Xpert”, “Xpert MTB/RIF”, ‘‘Cepheid”, ‘‘tuberculosis” and ‘‘*Mycobacterium* tuberculosis”.

### Study selection and data extraction

Two researchers (Shiying Li and Bin Liu) carried out the process of study-retrieval and data extraction independently. Any disagreements in the process were solved by discussing with a third researcher (Peng Hu).

#### Inclusion criteria

Inclusion criteria used in this meta-analysis were: (i) peer-reviewed, full-text, randomized controlled trials, cohort studies and cross-sectional studies, which used Xpert MTB/RIF for TB detection; (ii) specimens were tissues or body fluid collected from suspected TB patients; (iii) the number of cases ≥30; (iv) original data were sufficient to calculate the true positive (TP), false positive (FP), true negative (TN) and false negative (FN); (v) culture and/or a composite reference standard (CRS) was used as the reference standard in each individual study; and (vi) nationalities of individuals were clearly described.

#### Exclusion criteria

The initially selected articles were further screened based on the following exclusion criteria: (i) non-clinical research; (ii) abstract of any conference; (iii) case report; and (iv) review.

#### Data extraction

The basic characteristics of selected studies such as the year of publication, the number of the study population, number and type of samples, patients’ epidemiological and laboratory results, were collected. Additionally, the diagnostic characteristics of Xpert MTB/RIF such as TP, FP, TN, and FN were extracted. If data were insufficient in any study to perform a meta-analysis, we contacted the authors by e-mail for further information. If we were unable to obtain target data for certain studies, these studies were excluded.

### Imperfect reference standard

Culture is a gold standard for many infectious diseases except TB due to its paucibacillary characteristics, which may lead to a misdiagnosis of tested sample.[15] Assuming that Xpert MTB/RIF typically identifies TB in specimens with negative culture, this result would be considered as FP causing an underestimation of Xpert MTB/RIF’s true specificity. However, a CRS, which diagnoses the TB based on comprehensive results of clinical manifestations, signs and laboratory tests, might sometimes confirm the positivity of Xpert MTB/RIF for a sample with negative-TB culture and hence overestimate Xpert MTB/RIF’s accuracy. On the other hand, a CRS itself might reduce the accuracy of Xpert MTB/RIF by considering the result as FN.[16] Thus, to provide a more credible range of accuracy, we compared the accuracy of Xpert MTB/RIF to both the culture and CRS.

### Statistical analysis

MIDAS, a professional module of diagnostic test in STATA statistical software (version 12.0; STATA Corporation, College Station, TX, USA), was used to carry out the meta-analysis. The summary receiver operating characteristic model and bivariate random-effects model were carried out in this study to estimate the diagnostic accuracy of Xpert MTB/RIF. We calculated the sensitivity and specificity of Xpert MTB/RIF to diagnose TB for each individual study, then a pooled sensitivity, specificity, and area under the curve (AUC) were obtained, comparing with culture or CRS, along with 95% confidence intervals.

### Assessment of methodological qualities

The Review Manager software (version 5.3, The Nordic Cochrane Centre, Copenhagen, Denmark), which contains a Quality Assessment of Diagnostic Accuracy Studies-2 (QUADAS-2) tool, was used to evaluate the quality of selected studies. In QUADAS-2 tool, each question has three choices: yes, unclear or no. If1338 there was at least one ‘no’ or ‘unclear’ answer to a given question of a given domain, the risk for bias was considered as high or unclear, respectively.[17]

### Publication bias

In a systematic review, publication bias should be assessed to estimate whether the relevant studies with positive results are more likely to be published than the ones with negative results. However, there is no validated method for publication bias in test accuracy reviews yet. [18] So in this research, we did not estimate the publication bias.

### Heterogeneity analysis

A bivariate boxplot was used to assess the heterogeneity between included studies. It describes the degree of interdependence including the central location and identification of any outliers with an inner oval representing the median distribution of the data points and an outer oval representing the 95% confidence bound (by visually examining the position of each individual study, within the range of boxplot suggesting more heterogeneity). We predicted pre-existing heterogeneity in terms of sample types, patient age, status of HIV and smear-positivity. Therefore, studies were pre-specified into four subgroups: pulmonary versus extrapulmonary, adults versus children, HIV positive versus negative, and smear positive versus negative. Meta-analysis in each subgroup was only performed when at least four studies were available.[19]

## Results

### Description of studies

In total, we identified 106 studies (PRISMA flow chart and Supplementary reference) that included 52,410 samples for TB detection. All studies were in English except for two which were in Turkish. Among the 106 studies, 54 studies (47.8%) were carried out in 22 countries with high TB burden.[1]

The median number of specimens was 494 for TB detection. The proportions of HIV-positive patients ranged from 0% to 100%. In particular, two studies only included HIV patients, one study had no HIV patients and HIV status was unknown in 39 studies (S1 Table). Children were included in 21 studies, while patient’s age was unknown in another 30 studies (S1 Table). For sample type, pulmonary samples (such as sputum and bronchoalveolar lavage) were included in 71 studies, extrapulmonary samples (such as body fluid, fine needle aspiration, stool, and blood) were included in 25 studies and a mixture of pulmonary and extrapulmonary samples were used in 9 studies (S1 Table). The information of smear-positivity was reported in 44 included studies (S2 Table). The details of diagnostic accuracy of each individual study were shown in S2 Table.

### Methodological quality of selected studies

**Fig 1:**
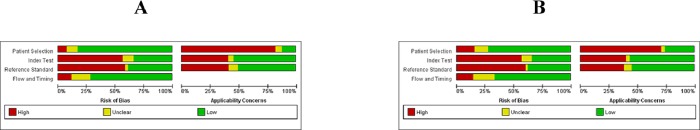
The overall methodological quality of all included studies for tuberculosis detection versus (A) culture reference standard, and (B) CRS.

The methodological qualities were estimated based on the culture and CRS. The overall methodological quality of included studies is summarized in Fig 1 (details of the quality assessment for each study are individually shown in S1 and S2 Figs). In the majority of studies, data was collected consecutively or randomly (n = 72; 67.9%) (S1 Table). All included studies were carried out either at reference laboratories or at tertiary care centers. Based on index tests, 11 studies (9.7%) were considered to carry an unclear risk of bias. Accordingly, the results of index test were interpreted blindly regardless of the reference standard results.

The result of heterogeneity analysis was shown in S3 Fig. A significant heterogeneity was found based on the bivariate box plot (culture, I^2^ = 99.90; CRS, I^2^ = 99.88).

### Sensitivities, specificities and AUCs of Xpert MTB/RIF for TB detection

**Fig 2:**
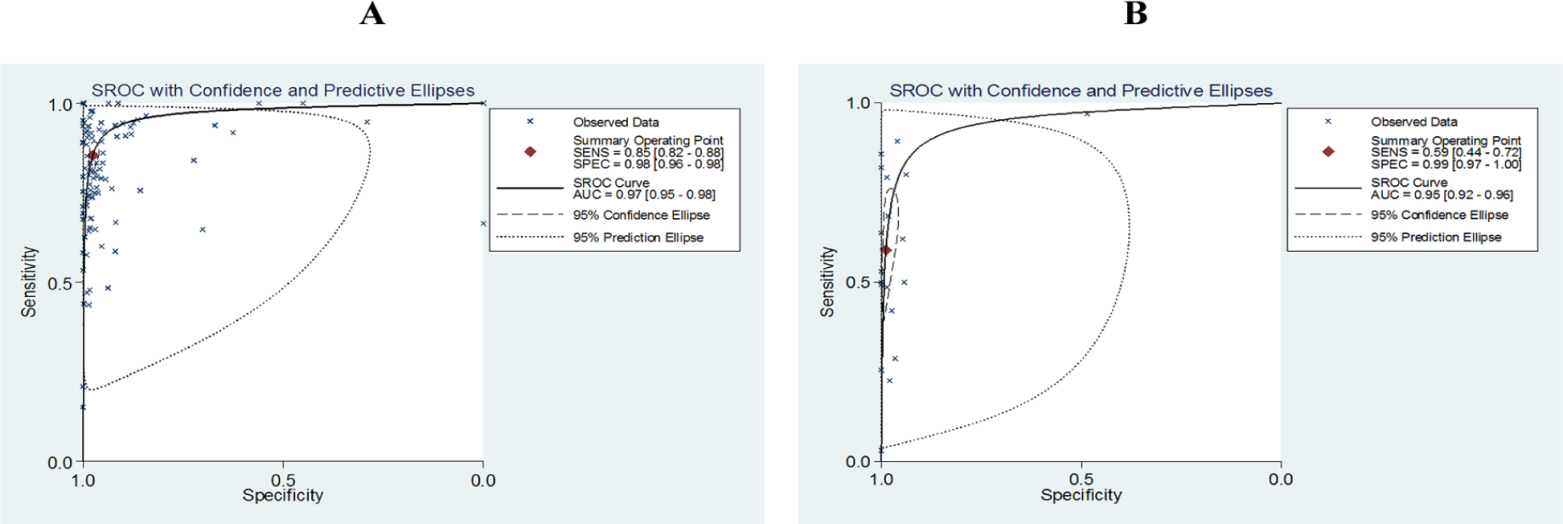
The SROC plot of Xpert MTB/RIF’s sensitivity, specificity and AUC (area under the curve) for tuberculosis detection versus (A) culture and (B) CRS. The sensitivity and specificity of one study were represented as a point; the summary sensitivity and specificity was represented as a summary point.

For the overall diagnostic accuracy of Xpert MTB/RIF, culture was used as the gold reference standard in 95 studies, while CRS was used in 20 studies. Pooled sensitivity, specificity and AUC were 0.85 (95% confidence interval [CI] 0.82–0.88), 0.98 (95% CI 0.96–0.98) and 0.97 (95% CI 0.95–0.98) compared to culture reference standard, respectively, while they were 0.59 (95% CI 0.44–0.77), 0.99 (95% CI 0.92–0.96) and 0.95 (95% CI 0.92–0.96) compared to CRS, respectively (Fig 2).

Since included studies were significantly heterogeneous, thus we further performed a separate meta-analysis for each of the four pre-identified subgroups based on the type of sample (pulmonary and extrapulmonary), subject’s age, HIV co-infection and smear-positivity. In the pulmonary and extrapulmonary subgroup, 74 studies included pulmonary samples while 26 studies included extrapulmonary samples. Pooled sensitivity, specificity and AUC of Xpert MTB/RIF for detecting pulmonary TB (PTB) were 0.87 (95% CI 0.83–0.90), 0.97 (95% CI 0.96–0.98) and 0.97 (95% CI 0.95–0.98) compared to culture reference standard, respectively; while they were 0.76 (95% CI 0.53–0.90), 0.97 (95% CI 0.87–1.00) and 0.95 (95% CI 0.92–0.96) compared to CRS, respectively. Likewise, pooled sensitivity, specificity and AUC of Xpert MTB/RIF for extrapulmonary TB (EPTB) detection were 0.80 (95% CI 0.69–0.88), 0.97 (95% CI 0.94–0.98) and 0.97 (95% CI 0.95–0.98) compared to culture reference standard, respectively; while they were 0.49 (95% CI 0.32–0.67), 0.99 (95% CI 0.97–1.00) and 0.94 (95% CI 0.92–0.96) compared to CRS, respectively (S4 Fig).

For different HIV co-infection status, diagnostic accuracy parameters could be collected on 26 studies and 18 studies that included HIV positive and negative cases, respectively. Pooled sensitivity, specificity and AUC of Xpert MTB/RIF for detecting TB in HIV positive patients were 0.81 (95% CI 0.73–0.87), 0.98 (95% CI 0.96–0.99) and 0.96 (95% CI 0.94–0.97) comparted to culture reference standard, respectively; while they were 0.63 (95% CI 0.45–0.87), 0.94 (95% CI 0.87–0.97) and 0.94 (95% CI 0.92–0.96) compared to CRS, respectively. Likewise, pooled sensitivity, specificity and AUC of Xpert MTB/RIF for detecting TB in HIV negative patients were 0.77 (95% CI 0.67–0.85), 0.99 (95% CI 0.97–0.99) and 0.98 (95% CI 0.96–0.99) comparted to culture reference standard, respectively; while they were 0.44 (95% CI 0.08–0.87), 0.99 (95% CI 0.93–1.00) and 0.98 (95% CI 0.97–0.99) compared to CRS, respectively (S5 Fig).

For smear-positivity status, samples of 38 studies were positive while they were negative in 43 studies. Compared to culture reference standard, pooled sensitivity, specificity and AUC of Xpert MTB/RIF for detecting TB in patients with positive smears were 0.99 (95% CI 0.97–0.99), 0.88 (95% CI 0.76–0.94) and 0.99 (95% CI 0.98–1.00), respectively; while they were 0.70 (95% CI 0.64–0.75), 0.98 (95% CI 0.96–0.99) and 0.89 (95% CI 0.86–0.929) in patients with negative smears, respectively. Similarly, compared to the CRS, Xpert MTB/RIF’s pooled sensitivity, specificity and AUC of for TB detection in patients with negative smears were 0.52 (95% CI 0.41–0.63), 0.99 (95% CI 0.95–1.00) and 0.68 (95% CI 0.63–0.72), respectively (S6 Fig). We were unable to determine diagnostic accuracy parameters of Xpert MTB/RIF compared to CRS in patients with positive smears due to the lack of this information.

Diagnosis of TB in children is rather difficult due to its typical paucibacillary characteristics and the difficulty of collecting sputum sample. Microscopic examination has a little value in diagnosis TB in children. Culture methods have a greater benefit, yet have a highly variable sensitivity. Clinical diagnosis of children TB relies mainly on a combination of symptoms, radiological findings, and identification of a tuberculosis contact.[20] Thus, the assessment of diagnostic accuracy of Xpert MTB/RIF for detecting TB in children is importantly needed. In the subgroup of subject’s age, there were 48 studies that included adults while 18 studies included children. Compared to culture reference standard, pooled sensitivity, specificity and AUC of Xpert MTB/RIF for detecting TB in adults were 0.82 (95% CI 0.76–0.86), 0.98 (95% CI 0.96–0.99) and 0.97 (95% CI 0.95–0.98) respectively; while they were 0.76 (95% CI 0.70–0.81), 0.98 (95% CI 0.96–0.99) and 0.90 (95% CI 0.87–0.93) in children, respectively. Likewise, compared to CRS, pooled sensitivity, specificity and AUC of Xpert MTB/RIF’s for TB detection in adults were 0.52 (95% CI 0.35–0.69), 0.99 (95% CI 0.97–1.00) and 0.96 (95% CI 0.94–0.97), respectively; while they were 0.55 (95% CI 0.41–0.65), 0.99 (95% CI 0.97–1.00) and 0.92 (95% CI 0.89–0.94) in children, respectively (S7 Fig).

### Sensitivities, specificities and AUCs of Xpert MTB/RIF in TB detection at different endemic degree regions

**Fig 3:**
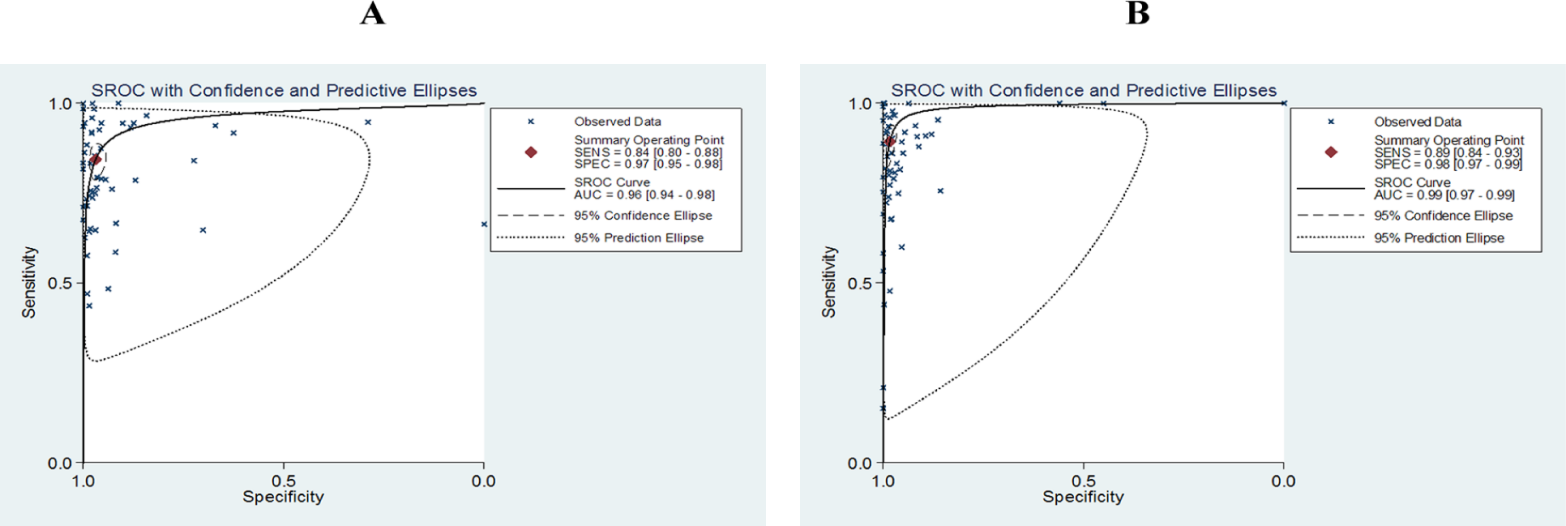
The SROC plot of Xpert MTB/RIF’s sensitivity, specificity and AUC (area under the curve) for tuberculosis detection in different prevalence regions versus culture reference standard. (A) High TB burden regions, (B) Middle and low prevalence regions. The sensitivity and specificity of one study were represented as a point; the summary sensitivity and specificity was represented as a summary point.

To evaluate the diagnostic accuracy of Xpert MTB/RIF assay in different regions, multiple meta-analyses were further carried out based on different TB prevalence. Since the data on CRS were limited within studies of different endemic regions, therefore all analyses were only relied on culture reference standard. As shown in Fig 3, 54 studies were carried out in 22 high TB burden countries while 56 studies were carried out in middle and low prevalence countries. Pooled sensitivity, specificity and AUC in highly endemic countries were 0.84 (95% CI 0.80–0.88), 0.97 (95% CI 0.95–0.98) and 0.96 (95% CI 0.94–0.98), respectively; while they were 0.89 (95% CI 0.84–0.93), 0.98 (95% CI 0.97–0.99) and 0.99 (95% CI 0.97–0.99) in countries with middle/low endemics, respectively.

Additionally, we sought to re-evaluate the diagnostic accuracy of Xpert MTB/RIF’s for the four subgroups among regions with different TB endemic level. However, data are quite limited for children; therefore, analyses were carried out considering the other 3 subgroups (sample’s type, HIV co-infection, and smear-positivity). For studies including PTB samples, pooled sensitivity, specificity and AUC were 0.84 (95% CI 0.80–0.88), 0.97 (95% CI 0.95–0.98) and 0.96 (95% CI 0.94–0.98) in high TB endemic countries, respectively; while they were 0.92 (95% CI 0.88–0.95), 0.98 (95% CI 0.96–0.99) and 0.99 (95% CI 0.97–0.99) in middle/low endemic countries, respectively (S8 Fig). For studies including EPTB samples, pooled sensitivity, specificity and AUC were 0.82 (95% CI 0.67–0.91), 0.92 (95% CI 0.84–0.96) and 0.94 (95% CI 0.92–0.96) in high TB endemic countries, respectively; while they were 0.81 (95% CI 0.63–0.91), 0.98 (95% CI 0.96–0.99) and 0.99 (95% CI 0.97–0.99) in middle/low endemic countries, respectively (S9 Fig).

For different HIV co-infection status, pooled sensitivity, specificity and AUC in high TB endemic countries were 0.80 (95% CI 0.75–0.84), 0.97 (95% CI 0.90–0.99) and 0.86 (95% CI 0.83–0.89) in HIV positive patients, respectively; while they were 0.75 (95% CI 0.66–0.82), 0.98 (95% CI 0.96–0.99) and 0.96 (95% CI 0.94–0.97) in HIV negative patients, respectively. Among middle/low TB endemic countries, pooled sensitivity, specificity and AUC were 0.81 (95% CI 0.61–0.92), 0.99 (95% CI 0.97–1.00) and 0.99 (95% CI 0.98–1.00) in HIV positive patient, respectively; while they were 0.76 (95% CI 0.47–0.92), 0.99 (95% CI 0.96–0.99) and 0.99 (95% CI 0.97–0.99) in HIV negative patients, respectively (S10 and S11 Fig).

Based on smear-positivity, pooled sensitivity, specificity and AUC in studies including positive smears were 0.97 (95% CI 0.95–0.98), 0.89 (95% CI 0.60–0.98) and 0.97 (95% CI 0.96–0.99) for countries with high TB endemic, respectively; while they were 0.99 (95% CI 0.97–1.00), 0.88 (95% CI 0.75–0.94) and 0.99 (95% CI 0.98–1.00) for countries with middle/low TB endemic, respectively (S12 Fig). For studies including negative smears, pooled sensitivity, specificity and AUC were 0.68 (95% CI 0.60–0.75), 0.96 (95% CI 0.90–0.98) and 0.84 (95% CI 0.81–0.87) for regions with high TB endemic, respectively; while they were 0.73 (95% CI 0.63–0.81), 0.99 (95% CI 0.97–1.00) and 0.93 (95% CI 0.91–0.95) for countries with middle/low TB endemic, respectively (S13 Fig).

## Discussion

Major advantages of this systemic review were the use of a pre-designed protocol, a comprehensive search, independent researchers, an effective model for meta-analysis (a bivariate random-effects model) and analyzing of four pre-identified subgroups to investigate the heterogeneity. In addition, effective communication with authors of studies with insufficient information resulted in a more efficient data analysis.

The comprehensive meta-analyses carried out in this systemic review revealed that Xpert MTB/RIF is a highly sensitive diagnostic tool for TB detection regardless sample’s type, status of HIV co-infection, subject’s age and smear-positivity. In addition, our analyses revealed that the sensitivity of Xpert MTB/RIF for TB detection compared to culture reference standard was higher than that compared to CRS (Table 1), which was consistent with previous studies.[21] Culture is an imperfect reference standard for TB detection, however, it requires more strict skills and long time. On the other hand, CRS is a composite of clinical investigations and laboratory examinations which offer more information to make a decision. However, there is a possibility that CRS could lead to a false positive diagnosis. Therefore, a combined application of the two reference standards (culture and CRS) may help to make more accurate clinical decisions.

**Table 1:** All the results of pooled sensitivities, specificities and AUCs of Xpert MTB/RIF for TB detection.

Meta-analysis		Culture			CRS		
		Sensitivity (95%IC)	Specificity (95%IC)	AUC (95%IC)	Sensitivity (95%IC)	Specificity (95%IC)	AUC (95%IC)
**Total**		**0.85(0.82–0.88)**	**0.98(0.96–0.98)**	**0.97(0.95–0.98)**	**0.59(0.44–0.72)**	**0.99(0.97–1.00)**	**0.95(0.92–0.96)**
Prevalence	High	0.84(0.80–0.88)	0.97(0.95–0.98)	0.96(0.94–0.98)	/	/	/
	Low&Middle	0.89(0.84–0.93)	0.98(0.97–0.99)	0.99(0.97–0.99)	/	/	/
**Pulmonary**		**0.87(0.83–0.90)**	**0.97(0.96–0.98)**	**0.97(0.95–0.98)**	**0.76(0.53–0.90)**	**0.97(0.87–1.00)**	**0.95(0.92–0.96)**
Prevalence	High	0.84(0.80–0.88)	0.97(0.95–0.98)	0.96(0.94–0.98)	/	/	/
	Low&Middle	0.92(0.88–0.95)	0.98(0.96–0.99)	0.99(0.97–0.99)	/	/	/
**Extra-pulmonary**		**0.80(0.69–0.88)**	**0.97(0.94–0.98)**	**0.97(0.95–0.98)**	**0.49(0.32–0.67)**	**0.99(0.97–1.00)**	**0.94(0.92–0.96)**
Prevalence	High	0.82(0.67–0.91)	0.92(0.84–0.96)	0.94(0.92–0.96)	/	/	/
	Low&Middle	0.81(0.63–0.91)	0.98(0.96–0.99)	0.99(0.97–0.99)	/	/	/
**Adults**		**0.82(0.76–0.86)**	**0.98(0.96–0.99)**	**0.97(0.95–0.98)**	**0.52(0.35–0.69)**	**0.99(0.97–1.00)**	**0.96(0.94–0.97)**
Prevalence	High	0.80(0.73–0.86)	0.96(0.92–0.98)	0.94(0.91–0.95)	/	/	/
	Low&Middle	0.82(0.72–0.89)	0.99(0.98–0.99)	0.98(0.97–0.99)	/	/	/
**Children**		**0.76(0.70–0.81)**	**0.98(0.96–0.99)**	**0.90(0.87–0.93)**	**0.55(0.41–0.65)**	**0.99(0.97–1.00)**	**0.92(0.89–0.94)**
Prevalence	High	0.75(0.69–0.79)	0.98(0.95–0.99)	0.87(0.64–0.90)	/	/	/
	Low&Middle	/	/	/	/	/	/
**HIV (+)**		**0.81(0.73–0.87)**	**0.98(0.96–0.99)**	**0.96(0.94–0.97)**	**0.63(0.45–0.77)**	**0.94(0.87–0.97)**	**0.94(0.92–0.96)**
Prevalence	High	0.80(0.75–0.84)	0.97(0.90–0.99)	0.86(0.83–0.89)	/	/	/
	Low&Middle	0.81(0.61–0.92)	0.99(0.97–1.00)	0.99(0.98–1.00)	/	/	/
**HIV (-)**		**0.77(0.67–0.85)**	**0.99(0.97–0.99)**	**0.98(0.96–0.99)**	**0.44(0.08–0.87)**	**0.99(0.93–1.00)**	**0.98(0.97–0.99)**
Prevalence	High	0.75(0.66–0.82)	0.98(0.96–0.99)	0.96(0.94–0.97)	/	/	/
	Low&Middle	0.76(0.47–0.92)	0.99(0.96–0.99)	0.99(0.97–0.99)	/	/	/
**Smear (+)**		**0.99(0.97–0.99)**	**0.88(0.76–0.94)**	**0.99(0.98–1.00)**	**/**	**/**	**/**
Prevalence	High	0.97(0.95–0.98)	0.89(0.60–0.98)	0.97(0.96–0.99)	/	/	/
	Low&Middle	0.99(0.97–1.00)	0.88(0.75–0.94)	0.99(0.98–1.00)	/	/	/
**Smear (-)**		**0.70(0.64–0.75)**	**0.98(0.96–0.99)**	**0.89(0.86–0.92)**	**0.52(0.41–0.63)**	**0.99(0.95–1.00)**	**0.68(0.63–0.72)**
Prevalence	High	0.68(0.60–0.75)	0.96(0.90–0.98)	0.84(0.81–0.87)	/	/	/
	Low&Middle	0.73(0.63–0.81)	0.99(0.97–1.00)	0.93(0.91–0.95)	/	/	/

**Abbreviations:** AUC: area under the curve.

Heterogeneity of diagnostic accuracy of Xpert MTB/RIF among all included studies was significantly higher when either compared to culture reference standard or CRS (Fig 1) indicating the variability of Xpert MTB/RIF’s diagnostic accuracy among different populations. Notably, the impact of sample type,[21,22] age of patients,[12] and status of HIV co-infection [23] and smear-positivity [24] are the main factors studied for their impact on Xpert MTB/RIF’s diagnostic accuracy. In this context, our results showed that Xpert MTB/RIF has higher sensitivity in patients with positive smears (0.99, 95% CI 0.97–0.99), in patients with PTB samples (0.87, 95% CI 0.83–0.90), in adults (0.82, 95% CI 0.76–0.86) and in HIV-positive patients (0.81, 95% CI 0.73–0.87). Overall, the sensitivities, specificities, and AUCs were ≥0.70, ≥0.97 and ≥89%, respectively (Table 1), indicating a high diagnostic accuracy of Xpert MTB/RIF for TB detection which is consistent with previous reports.[12,21,23,24] Given the extraordinary prevalence of TB worldwide since it is estimated that every year, nearly 10 million people fell ill with TB [1], Xpert MTB/RIF would be a perfect quick and accurate diagnostic assay for TB diagnosis. Notably, we found that Xpert MTB/RIF was more sensitive in HIV-positive than in HIV-negative individuals (81% versus 77%, compared to culture), which disagrees with the results of a previous review by Steingart et al., (HIV-positive versus HIV-negative: 79% versus 86%, compared to culture).[11] This difference could be attributed to the fact that Steingart et al addressed the accuracy of Xpert MTB/RIF only in the pulmonary samples of adults, while in our study, we addressed the overall diagnostic accuracy of Xpert MTB/RID regardless the sample type and subject’s age.

TB remains one of the world’s deadliest communicable diseases. There are some reviews that have addressed the accuracy of Xpert MTB/RIF either among specific population, such as adults [11] or in children,[12] or by testing specific samples, such as pulmonary,[11,12] or extrapulmonary samples.[21] Therefore, it is important to evaluate the overall diagnostic accuracy of Xpert MTB/RIF among countries with different levels of TB prevalence as well as different ages, sample sites, and HIV and smear statuses. To the best of our knowledge, our study was the first to investigate this issue. Based on our study, the overall pooled sensitivity of Xpert MTB/RIF for TB detection was lower in high TB prevalence countries than that of middle/low ones. Also, the same trend was found when different four subgroups were considered. Taken together, our results suggested that the diagnostic accuracy of Xpert MTB/RIF has a higher efficiency in countries with middle/low TB endemic burden than in countries with high endemic.

There are still some limitations in the current analysis such as there was no protocol available on how to handle non-respiratory specimens, specimen processing was extremely variable across studies and the CRS standard differed between studies. Therefore, the overall outcome should be interpreted with caution.

In conclusion, based on our meta-analyses using a bivariate model and the sufficient number of specimens, the diagnostic accuracy of Xpert MTB/RIF for TB detection was quite high. The overall sensitivity of Xpert MTB/RIF was lower in high TB burden countries than that of the middle/low burden. The results obtained in the current study will significantly help the physicians especially those in high-risk regions to make their clinical decision.

## Ethics approval and consent to participate

The experimental protocol was established, according to the ethical guidelines of the Helsinki Declaration and was approved by the Human Ethics Committee of Department of Infectious Diseases, The Second Affiliated Hospital, Chongqing Medical University. Written informed consent was obtained from individual participants.

## Supporting information

S1 File Supplementary reference. (DOC)

S1 Table Characteristics of studies included in the meta-analysis for tuberculosis detection. (DOC)

S2 Table Data of diagnostic accuracy of the study included in the meta-analysis for tuberculosis detection. (DOC)

S1 Fig Methodological quality summary on the quality assessment for each included study versus culture reference standard. (TIF)

S2 Fig Methodological quality summary on the quality assessment for each included study versus composite reference standard (CRS). (TIF)

S3 Fig Heterogeneity analyses of included studies for tuberculosis detection versus (A) culture reference standard, (B) composite reference standard.
(TIF)

S4 Fig The SROC plot of Xpert MTB/RIF’s sensitivity, specificity and AUC (area under the curve) for tuberculosis detection in pulmonary and extrapulmonary samples. (A) PTB, culture reference standard, (B) EPTB, culture reference standard, (C) PTB, composite reference standard, (D) EPTB, composite reference standard. The point represents the sensitivity and specificity of one study; the summary point represents the summary sensitivity and specificity.
(TIF)

S5 Fig The SROC plot of Xpert MTB/RIF’s sensitivity, specificity and AUC (area under the curve) for tuberculosis detection in HIV positive and negative patients. (A) HIV (+), culture reference standard, (B) HIV (-), culture reference standard, (C) HIV (+), composite reference standard, (D) HIV (-), composite reference standard. The point represents the sensitivity and specificity of one study; the summary point represents the summary sensitivity and specificity.
(TIF)

S6 Fig The SROC plot of Xpert MTB/RIF’s sensitivity, specificity and AUC (area under the curve) for tuberculosis detection in smear positive and negative samples. (A) Smear (+), culture reference standard, (B) Smear (-), culture reference standard. The point represents the sensitivity and specificity of one study; the summary point represents the summary sensitivity and specificity.
(TIF)

S7 Fig The SROC plot of Xpert MTB/RIF’s sensitivity, specificity and AUC (area under the curve) for tuberculosis detection in adults and children. (A) Adults, culture reference standard, (B) Children, culture reference standard, (C) Adults, composite reference standard, (D) Children, composite reference standard. The point represents the sensitivity and specificity of one study; the summary point represents the summary sensitivity and specificity.
(TIF)

S8 Fig The SROC plot of Xpert MTB/RIF’s sensitivity, specificity and AUC (area under the curve) for tuberculosis detection in pulmonary samples versus culture reference standard. (A) in high TB burden countries, (B) in middle/low TB burden countries. The point represents the sensitivity and specificity of one study; the summary point represents the summary sensitivity and specificity.
(TIF)

S9 Fig The SROC plot of Xpert MTB/RIF’s sensitivity, specificity and AUC (area under the curve) for tuberculosis detection in extrapulmonary samples. (A) in high TB burden countries, (B) in middle/low TB burden countries. The point represents the sensitivity and specificity of one study; the summary point represents the summary sensitivity and specificity.
(TIF)

S10 Fig The SROC plot of Xpert MTB/RIF’s sensitivity, specificity and AUC (area under the curve) for tuberculosis detection in HIV positive patients versus reference standard. (A) in high TB burden countries, (B) in middle/low TB burden countries. The point represents the sensitivity and specificity of one study; the summary point represents the summary sensitivity and specificity.
(TIF)

S11 Fig The SROC plot of Xpert MTB/RIF’s sensitivity, specificity and AUC (area under the curve) for tuberculosis detection in HIV negative patients versus reference standard. (A) in high TB burden countries, (B) in middle/low TB burden countries. The point represents the sensitivity and specificity of one study; the summary point represents the summary sensitivity and specificity.
(TIF)

S12 Fig The SROC plot of Xpert MTB/RIF’s sensitivity, specificity and AUC (area under the curve) for tuberculosis detection in smear positive samples. (A) in high TB burden countries, (B) in middle/low TB burden countries. The point represents the sensitivity and specificity of one study; the summary point represents the summary sensitivity and specificity.
(TIF)

S13 Fig The SROC plot of Xpert MTB/RIF’s sensitivity, specificity and AUC (area under the curve) for tuberculosis detection in smear negative samples. (A) in high TB burden countries, (B) in middle/low TB burden countries. The point represents the sensitivity and specificity of one study; the summary point represents the summary sensitivity and specificity.
(TIF)

S1 Text PRISMA flow chart. (TIF)

S2 Text PRISMA checklist. (DOC)
