# Supplementary material for: Diagnostic accuracy of Xpert MTB/RIF for tuberculosis detection in different regions with different endemic burden: A systematic review and meta-analysis
Source: PLoS One. 2017 Jul 14;12(7):e0180725. doi: 10.1371/journal.pone.0180725 (PMC5510832; doi:10.1371/journal.pone.0180725)
Supplement: S1 File — (DOC) [file pone.0180725.s001.doc]

**Diagnostic accuracy of Xpert** **MTB/RIF for tuberculosis detection in different regions with different endemic burden: a systematic review and meta-analysis**

Shiying Li^1^, Bin Liu^1^, Mingli Peng^1^, Min Chen^1^, Wenwei Yin^1^, Hui Tang^1^, Yuxuan Luo^1^, Peng Hu^1*^, and Hong Ren^1*^

1. Key Laboratory of Molecular Biology for Infectious Diseases (Ministry of Education), Institute for Viral Hepatitis, Department of Infectious Diseases, The Second Affiliated Hospital, Chongqing Medical University, Chongqing, PR China.

* Corresponding author at: Hong Ren and Peng Hu, Department of Infectious Diseases, The Second Affiliated Hospital, Chongqing Medical University, 74# Linjiang Road, Chongqing 400010, China. Tel: +86-2363693029, Fax: +86-2363703790. E-mail: renhong0531@vip.sina.com, hp_cq@163.com

**Supplementary reference**

1. Abed Al-Darraji HA, Abd Razak H, Ng KP, Altice FL, Kamarulzaman A. The diagnostic performance of a single GeneXpert MTB/RIF assay in an intensified tuberculosiscase finding survey among HIV-infected prisoners in Malaysia. *PLoS One* Sep **9**;8(9):e73717 (2013).

2. Ablanedo-Terrazas Y, Alvarado-de la Barrera C, Hernández-Juan R, Ruiz-Cruz M, Reyes-Terán G. Xpert MTB/RIF for diagnosis of tuberculous cervical lymphadenitis in HIV-infected patients. *Laryngoscope* Jun;**124**(6):1382-5 (2014).

3. Al-Ateah SM, Al-Dowaidi MM, El-Khizzi NA. Evaluation of direct detection of Mycobacterium tuberculosis complex in respiratory and non-respiratory clinical specimens using the Cepheid Gene Xpert® system. *Saudi Med J* Oct;**33**(10):1100-5 (2012).

4. Alvarez GG et al. The Feasibility, Accuracy, and Impact of Xpert MTB/RIF Testing in a Remote Aboriginal Community in Canada. *Chest* Sep **1**;148(3):767-73 (2015).

5. Antonenka U et al. Comparison of Xpert MTB/RIF with ProbeTec ET DTB and COBAS TaqMan MTB for direct detection of M. tuberculosis complex in respiratory specimens. *BMC Infect Dis* Jun **20**;13:280 (2013).

6. Atehortúa S, Ramírez F, Echeverri LM, Peñata A, Ospina S. Xpert MTB/RIF test performance assay in respiratory samples at real work settings in a developing country. *Biomedica* Mar;**35**(1):125-30 (2015).

7. Bablishvili N, Tukvadze N, Avaliani Z, Blumberg HM, Kempker RR.A comparison of the Xpert(®) MTB/RIF and GenoType(®) MTBDRplus assays in Georgia. *Int J Tuberc Lung Dis* Jun;**19**(6):676-8 (2015).

8. Balcells ME et al. Rapid molecular detection of pulmonary tuberculosis in HIV-infected patients in Santiago, Chile. *Int J Tuberc Lung Dis* Oct;**16**(10):1349-53 (2012).

9. Balcha TT et al. Intensified tuberculosis case-finding in HIV-positive adults managed at Ethiopian health centers: diagnostic yield of Xpert MTB/RIF compared with smear microscopy and liquid culture. *PLoS One* Jan **22**;9(1):e85478 (2014).

10. Barmankulova A, Higuchi M, Sarker MA, Alim MA, Hamajima N. Tuberculosis and rifampicin resistance among migrants in kyrgyzstan: detection by a new diagnostic test. *Nagoya J Med Sci* Feb;**77**(1-2):41-9 (2015).

11. Barnard M, Gey van Pittius NC, van Helden PD, Bosman M, Coetzee G, Warren RM. The diagnostic performance of the GenoType MTBDRplus version 2 line probe assay is equivalent to that of the Xpert MTB/RIF assay. *J Clin Microbiol* Nov;**50**(11):3712-6 (2012).

12. Bates M et al. Use of the Xpert(®) MTB/RIF assay for diagnosing pulmonary tuberculosis comorbidity and multidrug-resistant TB in obstetrics and gynaecology inpatient wards at the University Teaching Hospital, Lusaka, Zambia. *Trop Med Int Health* Sep;**18**(9):1134-40 (2013).

13. Bates M et al. Assessment of the Xpert MTB/RIF assay for diagnosis of tuberculosis with gastric lavage aspirates in children in sub-Saharan Africa: a prospective descriptive study. *Lancet Infect Dis* Jan;**13**(1):36-42 (2013).

14. Biadglegne F, Mulu A, Rodloff AC, Sack U. Diagnostic performance of the Xpert MTB/RIF assay for tuberculous lymphadenitis on fine needle aspirates from Ethiopia. *Tuberculosis (Edinb)* Sep;**94**(5):502-5 (2014).

15. Biadglegne F, Rodloff AC, Sack U. A first insight into high prevalence of undiagnosed smear-negative pulmonary tuberculosis in Northern Ethiopian prisons: implications for greater investment and quality control. *PLoS One* Sep **9**;9(9):e106869 (2014).

16. Blakemore R et al. Evaluation of the analytical performance of the Xpert MTB/RIF assay. *J Clin Microbiol* Jul;**48**(7):2495-501 (2010).

17. Boehme CC et al. Rapid molecular detection of tuberculosis and rifampin resistance. *N Engl J Med* Sep **9**;363(11):1005-15 (2010).

18. Boehme CC et al. Feasibility, diagnostic accuracy, and effectiveness of decentralised use of the Xpert MTB/RIF test for diagnosis of tuberculosis and multidrug resistance: a multicentre implementation study. *Lancet* Apr **30**;377(9776):1495-505 (2011).

19. Bowles EC, Freyée B, van Ingen J, Mulder B, Boeree MJ, van Soolingen D. Xpert MTB/RIF®, a novel automated polymerase chain reaction-based tool for the diagnosis oftuberculosis. *Int J Tuberc Lung Dis* Jul;**15**(7):988-9 (2011).

20. Carriquiry G et al. A diagnostic accuracy study of Xpert®MTB/RIF in HIV-positive patients with high clinical suspicion of pulmonary tuberculosis in Lima, Peru. *PLoS One*;**7**(9):e44626 (2012).

21. Causse M, Ruiz P, Gutiérrez-Aroca JB, Casal M. Comparison of two molecular methods for rapid diagnosis of extrapulmonary tuberculosis. *J Clin Microbiol* Aug;**49**(8):3065-7 (2011).

22. Chaisson LH et al. Impact of GeneXpert MTB/RIF assay on triage of respiratory isolation rooms for inpatients with presumed tuberculosis: a hypothetical trial. *Clin Infect Dis* Nov **15**;59(10):1353-60 (2014).

23. Chisti MJ et al. A prospective study of the prevalence of tuberculosis and bacteraemia in Bangladeshi children with severe malnutrition and pneumonia including an evaluation of Xpert MTB/RIF assay. *PLoS One* Apr **2**;9(4):e93776 (2014).

24. Ciftçi IH, Aslan MH, Aşık G. [Evaluation of Xpert MTB/RIF results for the detection of Mycobacterium tuberculosis in clinical samples]. *Mikrobiyol Bul* Jan;**45**(1):43-7 (2011). *Turkish*.

25. Coetzee L et al. Rapid diagnosis of pediatric mycobacterial lymphadenitis using fine needle aspiration biopsy. *Pediatr Infect Dis J* Sep;**33**(9):893-6 (2014).

26. Coleman M et al. Markers to differentiate between Kaposi's sarcoma and tuberculous pleural effusions in HIV-positive patients. *J Int J Tuberc Lung Dis* Feb;**19**(2):144-50 (2015).

27. Darban-Sarokhalil D et al. Comparison of smear microscopy, culture, and real-time PCR for quantitative detection of Mycobacterium tuberculosis in clinical respiratory specimens. *Scand J Infect Dis* Apr;**45**(4):250-5 (2013).

28. Deggim V, Somoskovi A, Voit A, Böttger EC, Bloemberg GV. Integrating the Xpert MTB/RIF assay into a diagnostic workflow for rapid detection of Mycobacteriumtuberculosis in a low-prevalence area. *J Clin Microbiol* Jul;**51**(7):2396-9 (2013).

29. Dorman SE et al. Performance characteristics of the Cepheid Xpert MTB/RIF test in a tuberculosis prevalence survey. *PLoS One*;**7**(8):e43307 (2012).

30. Du J et al. Rapid diagnosis of pleural tuberculosis by Xpert MTB/RIF assay using pleural biopsy and pleural fluid specimens. *J Res Med Sci* Jan;**20**(1):26-31 (2015).

31. Feasey NA et al. Evaluation of Xpert MTB/RIF for detection of tuberculosis from blood samples of HIV-infected adults confirms Mycobacterium tuberculosis bacteremia as an indicator of poor prognosis. *J Clin Microbiol* Jul;**51**(7):2311-6 (2013).

32. Friedrich SO et al. Assessment of the sensitivity and specificity of Xpert MTB/RIF assay as an early sputum biomarker of response to tuberculosis treatment. *Lancet Respir Med* Aug;**1**(6):462-70 (2013).

33. Giang do C et al. Prospective evaluation of GeneXpert for the diagnosis of HIV- negative pediatric TB cases. *BMC Infect Dis* Feb **18**;15:70 (2015).

34. Gu Y et al. Xpert MTB/RIF and GenoType MTBDRplus assays for the rapid diagnosis of bone and jointtuberculosis. *Int J Infect Dis* Jul;**36**:27-30 (2015).

35. Hanrahan CF et al. Xpert MTB/RIF as a measure of sputum bacillary burden. Variation by HIV status and immunosuppression. *Am J Respir Crit Care Med* Jun **1**;189(11):1426-34 (2014).

36. Helb D et al. Rapid detection of Mycobacterium tuberculosis and rifampin resistance by use of on-demand, near-patient technology. *J Clin Microbiol* Jan;**48**(1):229-37 (2010).

37. Hillemann D, Rüsch-Gerdes S, Boehme C, Richter E. Rapid molecular detection of extrapulmonary tuberculosis by the automated GeneXpert MTB/RIF system. *J Clin Microbiol* Apr;**49**(4):1202-5 (2011).

38. Huh HJ, Jeong BH, Jeon K, Koh WJ, Ki CS, Lee NY. Performance evaluation of the Xpert MTB/RIF assay according to its clinical application. *BMC Infect Dis* Nov **14**;14:589 (2014).

39. Hu P et al. Evaluation of the Xpert MTB/RIF assay for diagnosis of tuberculosis and rifampin resistance in county-level laboratories in Hunan province, China. *Chin Med J (Engl)* Nov;**127**(21):3744-50 (2014).

40. Iram S, Zeenat A, Hussain S, Wasim Yusuf N, Aslam M. Rapid diagnosis of tuberculosis using Xpert MTB/RIF assay - Report from a developing country. *Pak J Med Sci* Jan-Feb;**31**(1):105-10 (2015).

41. Ismail NA et al. Performance of a Novel Algorithm Using Automated Digital Microscopy for Diagnosing Tuberculosis. *Am J Respir Crit Care Med* Jun **15**;191(12):1443-9 (2015).

42. Jafari C, Ernst M, Kalsdorf B, Lange C. Comparison of molecular and immunological methods for the rapid diagnosis of smear-negativetuberculosis. *Int J Tuberc Lung Dis* Nov;**17**(11):1459-65 (2013).

43. Khalil KF, Butt T. Diagnostic yield of Bronchoalveolar Lavage gene Xpert in smear-negative and sputum-scarce pulmonary tuberculosis. *J Coll Physicians Surg Pak* Feb;**25**(2):115-8 (2015).

44. Kim CH et al. A comparison between the efficiency of the Xpert MTB/RIF assay and nested PCR in identifying Mycobacterium tuberculosis during routine clinical practice. *J Thorac Dis* Jun;**6**(6):625-31 (2014).

45. Kim CH et al. Identification of Mycobacterium tuberculosis and rifampin resistance in clinical specimens using theXpert MTB/RIF assay. *Ann Clin Lab Sci* Winter;**45**(1):32-8 (2015).

46. Kim MJ, Nam YS, Cho SY, Park TS, Lee HJ. Comparison of the Xpert MTB/RIF Assay and Real-time PCR for the Detection of Mycobacteriumtuberculosis. *Ann Clin Lab Sci* Spring;**45**(3):327-32 (2015).

47. Kim SY et al. The Xpert® MTB/RIF assay evaluation in South Korea, a country with an intermediate tuberculosisburden. *Int J Tuberc Lung Dis* Nov;**16**(11):1471-6 (2012).

48. Kim YW et al. Accuracy of the Xpert® MTB/RIF assay for the diagnosis of extra-pulmonary tuberculosis in South Korea. *Int J Tuberc Lung Dis* Jan;**19**(1):81-6 (2015).

49. Kokuto H, Sasaki Y, Yoshimatsu S, Mizuno K, Yi L, Mitarai S. Detection of Mycobacterium tuberculosis (MTB) in Fecal Specimens From Adults Diagnosed With Pulmonary Tuberculosis Using the Xpert MTB/Rifampicin Test. *Open Forum Infect Dis* May **22**;2(2):ofv074 (2015).

50. Kurbatova EV et al. Performance of Cepheid ® Xpert MTB/RIF ® and TB-Biochip ® MDR in two regions of Russia with a high prevalence of drug-resistant tuberculosis. *Eur J Clin Microbiol Infect Dis* Jun;**32**(6):735-43 (2013).

51. Kwak N et al. Diagnostic accuracy and turnaround time of the Xpert MTB/RIF assay in routine clinical practice. *PLoS One* Oct **29**;8(10):e77456 (2013).

52. LaCourse SM et al. Use of Xpert for the diagnosis of pulmonary tuberculosis in severely malnourished hospitalized Malawian children. *Pediatr Infect Dis J* Nov;**33**(11):1200-2 (2014).

53. Lawn SD et al. Screening for HIV-associated tuberculosis and rifampicin resistance before antiretroviral therapy using the Xpert MTB/RIF assay: a prospective study. *PLoS Med* Jul;**8**(7):e1001067 (2011).

54. Lawn SD, Kerkhoff AD, Vogt M, Wood R. Diagnostic accuracy of a low-cost, urine antigen, point-of-care screening assay for HIV-associated pulmonary tuberculosis before antiretroviral therapy: a descriptive study. *Lancet Infect Dis* Mar;**12**(3):201-9 (2012).

55. Lee HY et al. Diagnostic accuracy of Xpert® MTB/RIF on bronchoscopy specimens in patients with suspected pulmonary tuberculosis. *Int J Tuberc Lung Dis* Jul;**17**(7):917-21 (2013).

56. Le Palud P et al. Retrospective observational study of diagnostic accuracy of the Xpert® MTB/RIF assay on fiberoptic bronchoscopy sampling for early diagnosis of smear-negative or sputum-scarce patients with suspected tuberculosis. *BMC Pulm Med* Aug **12**;14:137 (2014).

57. Ligthelm LJ et al. Xpert MTB/RIF for rapid diagnosis of tuberculous lymphadenitis from fine-needle-aspiration biopsy specimens. *J Clin Microbiol* Nov;**49**(11):3967-70 (2011).

58. Lusiba JK et al. Evaluation of Cepheid's Xpert MTB/Rif test on pleural fluid in the diagnosis of pleural tuberculosisin a high prevalence HIV/TB setting. *PLoS One* Jul **22**;9(7):e102702 (2014).

59. Malbruny B, Le Marrec G, Courageux K, Leclercq R, Cattoir V. Rapid and efficient detection of Mycobacterium tuberculosis in respiratory and non-respiratory samples. *Int J Tuberc Lung Dis* Apr;**15**(4):553-5 (2011).

60. Marlowe EM et al. Evaluation of the Cepheid Xpert MTB/RIF assay for direct detection of Mycobacterium tuberculosiscomplex in respiratory specimens. *J Clin Microbiol* Apr;**49**(4):1621-3 (2011).

61. Meldau R et al. Comparison of same day diagnostic tools including Gene Xpert and unstimulated IFN-γ for the evaluation of pleural tuberculosis: a prospective cohort study. *BMC Pulm Med* Apr **8**;14:58 (2014).

62. Miller MB, Popowitch EB, Backlund MG, Ager EP. Performance of Xpert MTB/RIF RUO assay and IS6110 real-time PCR for Mycobacteriumtuberculosis detection in clinical samples. *J Clin Microbiol* Oct;**49**(10):3458-62 (2011).

63. Moure R, Martín R, Alcaide F. Effectiveness of an integrated real-time PCR method for detection of the Mycobacteriumtuberculosis complex in smear-negative extrapulmonary samples in an area of low tuberculosisprevalence. *J Clin Microbiol* Feb;**50**(2):513-5 (2012).

64. Moure R, Muñoz L, Torres M, Santin M, Martín R, Alcaide F. Rapid detection of Mycobacterium tuberculosis complex and rifampin resistance in smear-negative clinical samples by use of an integrated real-time PCR method. *J Clin Microbiol* Mar;**49**(3):1137-9 (2011).

65. Myneedu VP et al. Xpert(®) MTB/RIF assay for tuberculosis diagnosis: evaluation in an Indian setting. *Int J Tuberc Lung Dis* Aug;**18**(8):958-60 (2014).

66. Nhu NT et al. Evaluation of Xpert MTB/RIF and MODS assay for the diagnosis of pediatric tuberculosis. *BMC Infect Dis* Jan **23**;13:31 (2013).

67. Nhu NT et al. Evaluation of GeneXpert MTB/RIF for diagnosis of tuberculous meningitis. *J Clin Microbiol* Jan;**52**(1):226-33 (2014).

68. Nicol MP et al. Accuracy of the Xpert MTB/RIF test for the diagnosis of pulmonary tuberculosis in children admitted to hospital in Cape Town, South Africa: a descriptive study. *Lancet Infect Dis* Nov;**11**(11):819-24 (2011).

69. Nicol MP et al. Xpert MTB/RIF testing of stool samples for the diagnosis of pulmonary tuberculosis in children. *Clin Infect Dis* Aug;**57**(3):e18-21 (2013).

70. Ntinginya EN et al. Performance of the Xpert® MTB/RIF assay in an active case-finding strategy: a pilot study from Tanzania. *Int J Tuberc Lung Dis* Nov;**16**(11):1468-70 (2012).

71. Ou X et al. A feasibility study of the Xpert MTB/RIF test at the peripheral level laboratory in China. *Int J Infect Dis* Feb;**31**:41-6 (2015).

72. O'Grady J et al. Evaluation of the Xpert MTB/RIF assay at a tertiary care referral hospital in a setting wheretuberculosis and HIV infection are highly endemic. *Clin Infect Dis* Nov;**55**(9):1171-8 (2012).

73. Ozkutuk N, Surucüoglu S. Mikrobiyol Bul. [Evaluation of the Xpert MTB/RIF assay for the diagnosis of pulmonary and extrapulmonarytuberculosis in an intermediate-prevalence setting]. *Mikrobiyol Bul* Apr;**48**(2):223-32 (2014). *Turkish.*

74. Pandie S et al. Diagnostic accuracy of quantitative PCR (Xpert MTB/RIF) for tuberculous pericarditis compared to adenosine deaminase and unstimulated interferon-γ in a high burden setting: a prospective study. *BMC Med* Jun **18**;12:101 (2014).

75. Pang Y, Wang Y, Zhao S, Liu J, Zhao Y, Li H. Evaluation of the Xpert MTB/RIF assay in gastric lavage aspirates for diagnosis of smear-negative childhood pulmonary tuberculosis. *Pediatr Infect Dis J* Oct;**33**(10):1047-51 (2014).

76. Park KS et al. Comparison of the Xpert MTB/RIF and Cobas TaqMan MTB assays for detection of Mycobacteriumtuberculosis in respiratory specimens. *J Clin Microbiol* Oct;**51**(10):3225-7 (2013).

77. Patel VB et al. Diagnostic accuracy of quantitative PCR (Xpert MTB/RIF) for tuberculous meningitis in a high burden setting: a prospective study. *PLoS Med* Oct;**10**(10):e1001536 (2013).

78. Patel VB et al. Comparison of amplicor and GeneXpert MTB/RIF tests for diagnosis of tuberculous meningitis. *J Clin Microbiol* Oct;**52**(10):3777-80 (2014).

79. Peter J et al. Test characteristics and potential impact of the urine LAM lateral flow assay in HIV-infected outpatients under investigation for TB and able to self-expectorate sputum for diagnostic testing. *BMC Infect Dis* Jul **9**;15:262 (2015).

80. Porcel JM, Palma R, Valdés L, Bielsa S, San-José E, Esquerda A. Xpert® MTB/RIF in pleural fluid for the diagnosis of tuberculosis. *Int J Tuberc Lung Dis* Sep;**17**(9):1217-9 (2013).

81. Peter JG, Theron G, Muchinga TE, Govender U, Dheda K. The diagnostic accuracy of urine-based Xpert MTB/RIF in HIV-infected hospitalized patients who are smear-negative or sputum scarce. *PLoS One*;**7**(7):e39966 (2012).

82. Pinyopornpanish K et al. Comparison of Xpert MTB/RIF Assay and the Conventional Sputum Microscopy in Detecting Mycobacterium tuberculosis in Northern Thailand. *Tuberc Res Treat* **2015**:571782 (2015).

83. Rachow A et al. Rapid and accurate detection of Mycobacterium tuberculosis in sputum samples by Cepheid XpertMTB/RIF assay--a clinical validation study. *PLoS One* **6**(6):e20458 (2011).

84. Rachow A et al. Increased and expedited case detection by Xpert MTB/RIF assay in childhood tuberculosis: a prospective cohort study. *Clin Infect Dis* May;**54**(10):1388-96 (2012).

85. Reither K et al. Xpert MTB/RIF assay for diagnosis of pulmonary tuberculosis in children: a prospective, multi-centre evaluation. *J Infect* Apr;**70**(4):392-9 (2015).

86. Safianowska A, Walkiewicz R, Nejman-Gryz P, Grubek-Jaworska H. The use of selected commercial molecular assays for the microbiological diagnosis of tuberculosis. *Pneumonol Alergol Pol* **80**(1):6-12 (2012).

87. Sekadde MP et al. Evaluation of the Xpert MTB/RIF test for the diagnosis of childhood pulmonary tuberculosis in Uganda: a cross-sectional diagnostic study. *BMC Infect Dis* Mar **12**;13:133 (2013).

88. Darban-Sarokhalil D et al. Comparison of smear microscopy, culture, and real-time PCR for quantitative detection of Mycobacterium tuberculosis in clinical respiratory specimens. *Scand J Infect Dis* Apr;**45**(4):250-5 (2013).

89. Shah M et al. Comparative performance of urinary lipoarabinomannan assays and Xpert MTB/RIF in HIV-infected individuals. *AIDS* Jun **1**;28(9):1307-14 (2014).

90. Sohn H et al. Xpert MTB/RIF testing in a low tuberculosis incidence, high-resource setting: limitations in accuracy and clinical impact. *Clin Infect Dis* Apr;**58**(7):970-6 (2014).

91. Solomons RS et al. Improved diagnosis of childhood tuberculous meningitis using more than one nucleic acid amplification test. *Int J Tuberc Lung Dis* Jan;**19**(1):74-80 (2015).

92. Ssengooba W et al. Clinical utility of a novel molecular assay in various combination strategies with existing methods for diagnosis of HIV-related tuberculosis in Uganda. *PLoS One* Sep **15**;9(9):e107595 (2014).

93. Teo J, Jureen R, Chiang D, Chan D, Lin R. Comparison of two nucleic acid amplification assays, the Xpert MTB/RIF assay and the amplified Mycobacterium Tuberculosis Direct assay, for detection of Mycobacterium tuberculosis in respiratory and nonrespiratory specimens. *J Clin Microbiol* Oct;**49**(10):3659-62 (2011).

94. Theron G et al. Evaluation of the Xpert MTB/RIF assay for the diagnosis of pulmonary tuberculosis in a high HIV prevalence setting. *Am J Respir Crit Care Med* Jul **1**;184(1):132-40 (2011).

95. Theron G et al. Accuracy and impact of Xpert MTB/RIF for the diagnosis of smear-negative or sputum-scarcetuberculosis using bronchoalveolar lavage fluid. *Thorax* Nov;**68**(11):1043-51 (2013).

96. Theron G et al. Feasibility, accuracy, and clinical effect of point-of-care Xpert MTB/RIF testing for tuberculosis in primary-care settings in Africa: a multicentre, randomised, controlled trial. *Lancet* Feb **1**;383(9915):424-35 (2014).

97. Trajman A et al. Accuracy of polimerase chain reaction for the diagnosis of pleural tuberculosis. *Respir Med* Jun;**108**(6):918-23 (2014).

98. Tortoli E et al. Clinical validation of Xpert MTB/RIF for the diagnosis of extrapulmonary tuberculosis. *Eur Respir J* Aug;**40**(2):442-7 (2012).

99. Vadwai V, Boehme C, Nabeta P, Shetty A, Alland D, Rodrigues C. Xpert MTB/RIF: a new pillar in diagnosis of extrapulmonary tuberculosis? *J Clin Microbiol* Jul;**49**(7):2540-5 (2011).

100. van Kampen SC et al. Effects of Introducing Xpert MTB/RIF on Diagnosis and Treatment of Drug-Resistant TuberculosisPatients in Indonesia: A Pre-Post Intervention Study. *PLoS One* Jun **15**;10(6):e0123536 (2015).

101. Van Rie A et al. Diagnostic accuracy and effectiveness of the Xpert MTB/RIF assay for the diagnosis of HIV-associated lymph node tuberculosis. *Eur J Clin Microbiol Infect Dis* Nov;**32**(11):1409-15 (2013).

102. Williamson DA, Basu I, Bower J, Freeman JT, Henderson G, Roberts SA. An evaluation of the Xpert MTB/RIF assay and detection of false-positive rifampicin resistance in Mycobacterium tuberculosis. *Diagn Microbiol Infect Dis* Oct;**74**(2):207-9 (2012).

103. Yin QQ et al. Rapid diagnosis of childhood pulmonary tuberculosis by Xpert MTB/RIF assay using bronchoalveolar lavage fluid. *Biomed Res Int*;**2014**:310194 (2014).

104. Yoon C et al. Impact of Xpert MTB/RIF testing on tuberculosis management and outcomes in hospitalized patients in Uganda. *PLoS One* **7**(11):e48599 (2012).

105. Zar HJ et al. Rapid molecular diagnosis of pulmonary tuberculosis in children using nasopharyngeal specimens. *Clin Infect Dis* Oct;**55**(8):1088-95 (2012).

106. Zar HJ, Workman L, Isaacs W, Dheda K, Zemanay W, Nicol MP. Rapid diagnosis of pulmonary tuberculosis in African children in a primary care setting by use ofXpert MTB/RIF on respiratory specimens: a prospective study. *Lancet Glob Health* Aug;**1**(2):e97-104 (2013).
