# Supplementary material for: Diagnostic accuracy of Xpert MTB/RIF for tuberculosis detection in different regions with different endemic burden: A systematic review and meta-analysis
Source: PLoS One. 2017 Jul 14;12(7):e0180725. doi: 10.1371/journal.pone.0180725 (PMC5510832; doi:10.1371/journal.pone.0180725)
Supplement: S1 Table — (DOC) [file pone.0180725.s002.doc]

**Diagnostic accuracy of Xpert** **MTB/RIF for tuberculosis detection in different regions with different endemic burden: a systematic review and meta-analysis**

Shiying Li^1^, Bin Liu^1^, Mingli Peng^1^, Min Chen^1^, Wenwei Yin^1^, Hui Tang^1^, Yuxuan Luo^1^, Peng Hu^1*^, and Hong Ren^1*^

1. Key Laboratory of Molecular Biology for Infectious Diseases (Ministry of Education), Institute for Viral Hepatitis, Department of Infectious Diseases, The Second Affiliated Hospital, Chongqing Medical University, Chongqing, PR China.

* Corresponding author at: Hong Ren and Peng Hu, Department of Infectious Diseases, The Second Affiliated Hospital, Chongqing Medical University, 74# Linjiang Road, Chongqing 400010, China. Tel: +86-2363693029, Fax: +86-2363703790. E-mail: renhong0531@vip.sina.com, hp_cq@163.com

**Supplementary table 1.** Characteristics of studies included in the meta-analysis for tuberculosis detection.

| Study | First author Supplementary ref. | Year | Study setting | Male (%) | HIV (%) | Age (year)  (Median, IQR) | Patient selecting method | Total samples n (included n) | Specimen type (samples n) | Gold standard | Sample used for gold standard | Composite reference standard |
| --- | --- | --- | --- | --- | --- | --- | --- | --- | --- | --- | --- | --- |
| 1 | Abed Al-Darraji HA ^1^  Malaysia | 2013 | Clinical | 113  (90.4) | 125  (100) | 61.6%<40, 38.4%≥40 | Cross-sectional  Consecutive | 125  (125) | Sputum  (125) | Liquid culture | As for Xpert | None |
| 2 | Ablanedo-Terrazas Y ^2^  Mexico | 2014 | Clinical | 60  (88.2) | 68  (100) | Adult>16  (29, 24-35.5) | Prospective  Consecutive | 68  (68) | Lymph node specimens (68) | Culture | As for Xpert | None |
| 3 | Al-Ateah SM ^3^  Saudi Arabia | 2012 | Laboratory | 126  (53.8) | 1  (0.4) | NR | Cross-sectional  Unspecified | 234  (239) | Sputum (56), BAL (116); tissue (16), CSF (14), FNA (5), body fluid (22), abscess (10) | Solid and/or liquid culture | As for Xpert | None |
| 4 | Alvarez GG ^4^  Canada | 2015 | Laboratory | 220  (64) | NR | Median 36, 25-38 | Prospective  Consecutive | 344  (413) | Sputum  (344) | Culture | As for Xpert | None |
| 5 | Antonenka U ^5^  German | 2013 | Clinical | NR | NR | NR | Retrospective  Unspecified | 121  (121) | Respiratory specimens (121) | Solid or liquid culture | As for Xpert | None |
| 6 | Atehortúa S ^6^  Colombia | 2015 | Clinical | 45  (43.6) | 20  (19.4) | ≥12  (37.5, 27-49) | Cross-sectional  Unspecified | 103  (103) | BAL (63), Sputum (36), TA (4) | Ogawa-Kudoh culture | As for Xpert | None |
| 7 | Bablishvili N ^7^  Georgia | 2015 | Laboratory | NR | NR | NR | Unspecified  Unspecified | 366  (366) | Sputum (366) | Solid and/or liquid culture | As for Xpert | None |
| 8 | Balcells ME ^8^  Chile | 2012 | Clinical | 127  (79.4) | 160  (100) | Adults>18  (37.4, 19-65) | Cross-sectional  Prospective  Consecutive | 160  (160) | Sputum  (160) | Solid and liquid culture | As for Xpert | None |
| 9 | Balcha TT ^9^  Ethiopia | 2014 | Clinical | 476  (58.6) | 812  (100) | Adult≥18  (32, 28-40) | Prospective  Consecutive | 812  (135) | Sputum (812) | Liquid culture | As for Xpert | None |
| 10 | Barmankulova A ^10^  Kyrgyzstan | 2015 | Laboratory | 172  (57.3) | NR | Median 34, IQR 25-45 | Cross-sectional  Unspecified | 300  (291) | Sputum (300) | Solid and liquid culture | As for Xpert | None |
| 11 | Barnard M ^11^  South Africa | 2012 | Laboratory | NR | NR | NR | Unspecified  Consecutive | 282  (68) | Sputum (282) | Liquid culture | As for Xpert | None |
| 12 | Bates M ^12^  Zambia | 2013 | Clinical | 0  (0) | 62  (66.0) | Adult>20  (28, 24-33) | Prospective  Unspecified | 94  (94) | Sputum (94) | Liquid culture | As for Xpert | None |
| 13 | Bates M ^13^  Zambia | 2013 | Clinical | NR | 22  (2.4) | Children≤15 | Prospective  Unspecified | 930  (930) | Sputum, gastric lavage aspirate (930) | Liquid culture | As for Xpert | None |
| 14 | Biadglegne F ^14^  Ethiopia | 2014 | Clinical | 99  (42.9) | NR | 14.7%≤14, 85.3%>14 | Cross-sectional  Unspecified | 231  (220) | Lymph node aspirates (231) | Solid culture | As for Xpert | None |
| 15 | Biadglegne F ^15^  Ethiopia | 2014 | Laboratory | 195  (97.5) | NR | Median 39, IQR 13-73 | Cross-sectional  Consecutive | 200  (200) | Sputum (200) | Culture | As for Xpert | None |
| 16 | Blakemore R ^16^  America | 2010 | Clinical | NR | NR | NR | Unspecified  Unspecified | 168  (168) | Sputum (168) | Solid culture | As for Xpert | None |
| 17 | Boehme CC ^17^ | 2010 | Clinical | 929  (53.7) | 392  (22.7) | Adults≥18  (34, 17-88) | Prospective  Consecutive | 1,730  (1,357) | Sputum (1,730) | Liquid culture | As for Xpert | None |
|  | Peru |  |  | 181  (53.1) | 3  (0.9) | Adults≥18  (31, 18-79) |  | 341  (313) | Sputum (313) |  |  |  |
|  | Azerbaijan |  |  | 251  (71.1) | 9  (2.6) | Adults≥18  (37, 20-69) |  | 353  (219) | Sputum (353) |  |  |  |
|  | South Africa |  |  | 357  (49.2) | 376  (51.8) | Adults≥18  (34, 18-74) |  | 726  (601) | Sputum (726) |  |  |  |
|  | India |  |  | 140  (45.2) | 4  (12.9) | Adults≥18  (30, 17-88) |  | 310  (224) | Sputum (310) |  |  |  |
| 18 | Boehme CC ^18^ | 2011 | Clinical | 4,043  (60.8) | 1,255  (18.9) | Adults≥18  (38, 29-50) | Unspecified  Consecutive | 6,648  (3,909) | Sputum (6,648) | Solid or liquid culture | As for Xpert | None |
|  | Peru |  |  | 607  (51.2) | 5  (0.4) | Adults≥18  (37, 26-53) |  | 1,185  (1,005) | Sputum (1,185) | Liquid culture |  |  |
|  | Azerbaijan |  |  | 748  (99.9) | 1  (0.1) | Adults≥18  (36, 30-44) |  | 749  (536) | Sputum (749) | Solid and liquid culture |  |  |
|  | South Africa |  |  | 1,275  (50.6) | 947  (37.5) | Adults≥18  (36, 29-46) |  | 2,522  (904) | Sputum (2,522) | Solid and liquid culture |  |  |
|  | Uganda |  |  | 202  (54.3) | 254  (68.3) | Adults≥18  (32, 26-38) |  | 372  (289) | Sputum (289) | Solid culture |  |  |
|  | India |  |  | 628  (69.6) | 40  (4.4) | Adults≥18  (45, 32-58) |  | 902  (289) | Sputum (902) | Liquid an Ogawa liquid |  |  |
|  | Philippines |  |  | 583  (63.5) | 8  (0.9) | Adults≥18  (47, 34-58) |  | 918  (387) | Sputum (918) | Liquid culture |  |  |
| 19 | Bowles EC ^19^  Netherlands | 2011 | Clinical | NR | NR | NR | Unspecified  Unspecified | 89  (89) | Sputum (86), pleural fluid (1), gastric fluid (1), bronchial washing (1) | Liquid culture | As for Xpert | None |
| 20 | Carriquiry G ^20^  Peru | 2012 | Clinical | 95  (73) | 131  (100) | Adults≥18  (35, 29-42) | Cross-sectional  Unspecified | 131  (131) | Sputum (131) | Solid and liquid culture | As for Xpert | None |
| 21 | Causse M ^21^  Spain | 2011 | Clinical | 184  (63.8) | NR | Median 45 | Unspecified  Consecutive | 289  (340) | CSF (50), pleural fluid (34), articular fluid (58), ascitic fluid (20), biopsy specimens (98), gastric aspirates (54), pericardial fluid (12), and purulent exudates (14) | Solid or liquid culture | As for Xpert | None |
| 22 | Chaisson LH ^22^  America | 2014 | Clinical | 107  (77.0) | 42  (30.2) | Median 54, IQR 43-60 | Prospective  Consecutive | 139  (142) | Sputum (142) | Liquid culture | As for Xpert | None |
| 23 | Chisti MJ ^23^  Bangladesh | 2014 | Clinical | NR | NR | Children<5  (10 months, 5-18 months) | Prospective  Consecutive | 405  (214) | Sputum (214) | Solid culture | Sputum, Gastric  lavage | None |
| 24 | Ciftçi IH ^24^  Turkey | 2011 | Clinical | NR | NR | NR | Unspecified  Unspecified | 85  (85) | Sputum (50), BAL (25), thorasynthesis fluid (5), urine (5) | Liquid culture | As for Xpert | None |
| 25 | Coetzee L ^25^  South Africa | 2014 | Clinical | 43  (39.1) | 6  (5.5) | Children<13 | Prospective  Unspecified | 110  (72) | FNA (110) | Liquid culture | As for Xpert | Culture and/or cytomorphology |
| 26 | Coleman M ^26^  Malawi | 2015 | Clinical | 30  (60) | 50  (100) | Adult≥16  (32, 17-54) | Prospective  Consecutive | 50  (50) | Pleural effusion (50) | Liquid culture | As for Xpert | None |
| 27 | Darban-Sarokhalil D ^27^  Iran | 2013 | Laboratory | NR | NR | NR | Unspecified  Unspecified | 247  (247) | Sputum (247) | Solid culture | As for Xpert | None |
| 28 | Deggim V ^28^  Switzerland | 2013 | Clinical | NR | NR | NR | Prospective  Unspecified | 79  (77) | Sputum (57), bronchial aspirates (10) and BAL (4); Biopsies (3), CSF (1), ascitic fluid (1), pleural fluid (1), pus (1), and pharynx aspirate (1) | Liquid culture | As for Xpert | None |
| 29 | Dorman SE ^29^  South Africa | 2012 | Laboratory | 6,469  (93.8) | 602  (8.7) | Median 43, IQR 34-49 | Cross-sectional  Consecutive | 6,893  (6,621) | Sputum  (6,893) | Liquid culture | As for Xpert | None |
| 30 | Du J ^30^  China | 2015 | Clinical | 70  (55.6) | 5  (4.0) | Adults>16  (38.6, 25.4-51.8) | Unspecified  Unspecified | 126  (126) | Pleural biopsy (126), pleural fluid specimens (126) | Culture | As for Xpert | None |
| 31 | Feasey NA ^31^  Malawi | 2013 | Clinical | 69  (66.3) | 104  (100) | Median 36.9, 26.7-47.1 | Prospective  Consecutive | 104  (104) | Blood (104) | Solid culture | Sputum | None |
| 32 | Friedrich SO ^32^  South Africa  Tanzania | 2013 | Clinical | 144  (65) | 20  (9) | Median 30, IQR 23-40 | Unspecified  Random | 1,918  (1,918) | Sputum (1,918) | Solid and liquid culture | As for Xpert | Culture and smear microscopy |
| 33 | Giang do C ^33^  Vietnam | 2015 | Clinical | 98  (65.3) | 0  (0) | Children<15  (18.5 months, 5-170 months) | Prospective  Consecutive | 150  （150） | Sputum (79), Gastric fluid (215), CSF (3), Pleural fluid (4), Cervical lymphadenopathic pus (1) | Liquid culture | As for Xpert | Confirmed (clinical sign/symptom and smear/culture) or Probable (clinical sign/symptom and response to anti-TB therapy/exposure to TB case) or Possible (only clinical symptoms and/or signs suggestive of TB) or Not TB |
| 34 | Gu Y ^34^  China | 2015 | Clinical | 28  (46.7) | NR | Median 39.7, IQR 19.5-74.6 | Prospective  Unspecified | 60  (60) | Pus specimens (60) | Liquid culture | As for Xpert | Culture and/or histopathological evidence and/or response to anti-TB therapy |
| 35 | Hanrahan CF ^35^  South Africa | 2014 | Clinical | 907  (38) | 1,394  (58) | Adult≥15  (37, 29-45) | Cross-sectional  Consecutive | 2,406  (2,082) | Sputum (2,082) | Liquid culture | As for Xpert | None |
| 36 | Helb D ^36^ | 2010 | Clinical | 112  (65.5) | 21  (12.3) | Median 34, IQR 18-70 | Retrospective  Consecutive | 171  (191) | Sputum (191) | Solid or liquid culture | As for Xpert | None |
|  | Vietnam |  |  | 74  (69.2) | 1  (0.9) | Median 34, IQR 18-76 |  | 107  (107) | Sputum (107) |  |  |  |
|  | Uganda |  |  | 38  (59.3) | 20  (31.3) | Median 34, IQR 18-60 |  | 64  (84) | Sputum (84) |  |  |  |
| 37 | Hillemann D ^37^  German | 2011 | Laboratory | NR | NR | NR | Unspecified  Consecutive | 521  (477) | Urine (91), gastric aspirate (30), tissue (245), pleural fluid (113), CSF (19), stool (23) | Solid or liquid culture | As for Xpert | None |
| 38 | Huh HJ ^38^  South Korea | 2014 | Clinical | 197  (65.7) | 1  (0.3) | Median 58, IQR 18-93 | Retrospective  Consecutive | 300  (303) | Sputum (264), Bronchial washing or BAL (39) | Solid and liquid culture | As for Xpert | None |
| 39 | Hu P ^39^  China | 2014 | Laboratory | 1,037  (76.7) | NR | 3.2%<20, 96.8%≥20 | Unspecified  Consecutive | 1,352  (728) | Sputum (1,352) | Solid culture | As for Xpert | None |
| 40 | Iram S ^40^  Pakistan | 2015 | Clinical | NR | NR | NR | Prospective  Convenience | 245  (245) | Sputum (205), Pus (19), Pleural fluid (11), Ascitic fluid (7), Pericardial fluid (1), CSF (1), Urine (1) | Solid culture | As for Xpert | None |
| 41 | Ismail NA ^41^  South Africa | 2015 | Laboratory | NR | NR | Adult≥18 | Prospective  Consecutive | 404  (404) | Sputum (404) | Liquid culture | As for Xpert | None |
| 42 | Jafari C ^42^  Germany | 2013 | Clinical | 58  (60.4) | 3  (3.1) | Adult>17  Median 56.7 | Retrospective  Consecutive | 96  (96) | Sputum and BAL  (96) | Culture | As for Xpert | Culture or Clinical symptoms and imaging findings and/or histopathology and response to anti-TB treatment |
| 43 | Khalil KF ^43^  Pakistan | 2015 | Clinical | 36  (38.7) | 0  (0) | Adults>16,  (38.56, 19.5-57.6) | Unspecified  Consecutive | 93  (93) | BAL (93) | Solid culture | As for Xpert | None |
| 44 | Kim CH ^44^  South Korea | 2014 | Clinical | 104  (60.8) | 1  (0.6) | Median 58.6, IQR 41.02-76.18 | Retrospective  Unspecified | 171  (171) | Pulmonary (160), Non-pulmonary (38) specimens | Liquid culture | As for Xpert | None |
| 45 | Kim CH ^45^  South Korea | 2015 | Clinical | 217  (56.7) | 1  (0.3) | Median 56.31, IQR 38.43-74.18 | Retrospective  Convenience | 383  (444) | Sputum (176), Bronchial washes (225), BAL (4); Pleural fluid (36), Tissue (1), Pericardial fluid (1), Lymph node (1) | Liquid culture | As for Xpert | None |
| 46 | Kim MJ ^46^  South Korea | 2015 | Laboratory | NR | NR | NR | Unspecified Convenience | 52  (52) | Sputum (36), bronchial washing (10), pleural fluid (3), pleural mass (1), urine (2) | Solid and liquid culture | As for Xpert | None |
| 47 | Kim SY ^47^  South Korea | 2012 | Clinical | NR | NR | NR | Unspecified  Consecutive | 71  (69) | Sputum (71) | Solid and liquid culture | As for Xpert | None |
| 48 | Kim YW ^48^  South Korea | 2015 | Clinical | 761  (53.3) | 12  (0.8) | Median 59, IQR 44-71 | Retrospective  Consecutive | 1,429  (1,540) | LN and tissue/pus (397), body fluid (469), CSF (254), joint fluid (283), urine (106), others (31) | Liquid culture | As for Xpert | Confirmed(culture) or Probable (clinical symptoms, radiological findings and/or histology/cytology suggestive of TB) or Possible (only clinical symptoms and/or signs suggestive of TB) |
| 49 | Kokuto H ^49^  Japan | 2015 | Clinical | 51  (54.8) | 0  (0) | Adult≥20  (59.6, 45.0-75.0) | Retrospective  Convenience | 93  (93) | Fecal specimen s (93) | Liquid culture | Sputum | Culture and/or molecular diagnostics |
| 50 | Kurbatova EV ^50^  Russia | 2013 | Clinical | NR | NR | Adults≥18 | Unspecified  Consecutive | 201  (236) | Sputum (236) | Solid and liquid culture | As for Xpert | None |
| 51 | Kwak N ^51^  South Korea | 2013 | Clinical | 426  (62.5) | 5  (0.7) | Median 61, IQR 47.5-73.0 | Retrospective  Unspecified | 681  (661) | Sputum (681) | Culture | As for Xpert | None |
| 52 | LaCourse SM ^52^  Malawi | 2014 | Clinical | NR | 52  (17.3) | Median 18.5 months, IQR 12.1-25.6 months | Prospective  Consecutive | 300  (300) | Sputum (300) | Liquid culture | As for Xpert | None |
| 53 | Lawn SD ^53^  South Africa | 2011 | Clinical | 162  (34.6) | 468  (100) | Adults≥18  (33.6, 27.8-40.7) | Prospective  Consecutive | 468  (445) | Sputum (468) | Liquid culture | As for Xpert | None |
| 54 | Lawn SD ^54^  South Africa | 2012 | Clinical | 185  (36) | 516  (100) | Adults>18 | Prospective  Consecutive | 516  (516) | Sputum (516) | Liquid culture | As for Xpert | None |
| 55 | Lee HY ^55^  South Korea | 2013 | Clinical | 78  (59.1) | 1  (0.8) | Median 54.0, IQR 18-90 | Retrospective  Unspecified | 132  (132) | Bronchoscopy specimens (132) | Liquid culture | Sputum or bronchoscopy specimens | None |
| 56 | Le Palud P ^56^  France | 2014 | Clinical | 102  (63.0) | 7  (4.3) | Median 54, IQR 34-74 | Retrospective  Consecutive | 162  (162) | BA (48), BAL (47), BA/BAL mix (67) | Liquid culture | As for Xpert | None |
| 57 | Ligthelm LJ ^57^  South Africa | 2011 | Clinical | 20  (41.7) | 9  (18.8) | 16.7%≤20, 83.3%>20 | Unspecified  Unspecified | 48  (48) | Fine-needle-aspiration biopsy specimens (48) | Liquid culture | As for Xpert | None |
| 58 | Lusiba JK ^58^  Uganda | 2014 | Clinical | 66  (57) | 52  (44.8) | Adult≥18  (34, 21-47) | Unspecified  Consecutive | 116  (116) | Pleural fluid (116) | Solid culture | Pleural tissue | Culture and/or histopathology |
| 59 | Malbruny B ^59^  France | 2011 | Clinical | 79  (59.8) | NR | Median 52 | Prospective  Unspecified | 132  (180) | Gastric aspirate (33), bronchial aspirate (31), sputum (18), bronchoalveolar lavage (9); lymph node (23), CSF (15), pleural fluid (12), vertebral biopsy (6), purulent exudates (6), joint fluid (5), ascitic fluid (5), skin biopsy (5), lung biopsy (4), peritoneal fluid (3), urine (3), bone marrow (2) | Solid and/or liquid culture | As for Xpert | None |
| 60 | Marlowe EM ^60^  America | 2011 | Laboratory | NR | NR | NR | Unspecified  Random | 216  (216) | Sputum  (216) | Solid or liquid culture | As for Xpert | None |
| 61 | Meldau R ^61^  South Africa | 2014 | Clinical | 53  (60.2) | 9  (10.2) | Median 51.0, IQR 42.6-62.6 | Prospective  Consecutive | 88  (88) | Pleural fluid (93), Sputum (15), Pleural biopsy(92) | Liquid culture | As for Xpert | Culture and/or histopathology |
| 62 | Miller MB ^62^  America | 2011 | Laboratory | NR | NR | NR | Retrospective  Consecutive | 90  (112) | Sputum (69), bronchial brush (1), bronchial wash (6), biopsy specimen (13), nasopharyngeal aspirate (1), pleural fluid (3) ,TA (2) , BAL (14), abscess (1), retroperitoneal fluid (1), stool (1) | Solid and liquid culture | As for Xpert | None |
| 63 | Moure R ^63^  Spain | 2012 | Laboratory | NR | NR | NR | Unspecified  Unspecified | 149  (147) | Sterile fluid (58), nonsterile fluid (12), lymph nodes (38), abscess aspirates (19), tissue (20), stool (2) | Culture | As for Xpert | None |
| 64 | Moure R ^64^  Spain | 2011 | Clinical | NR | NR | NR | Retrospective  Unspecified | 122  (124) | Sputum (92), BA (12), pulmonary biopsy (1); pleural fluid (4), gastric aspirate (5), urine (2), stool (1),cerebrospinal fluid (3), ascitic fluid (2), lymph node aspirate (1), skin biopsy (1), mammary abscess (1) | Solid and liquid culture | As for Xpert | None |
| 65 | Myneedu VP ^65^  India | 2014 | Laboratory | NR | NR | NR | Unspecified  Unspecified | 134  (120) | Sputum  (134) | Solid and/or liquid culture | As for Xpert | None |
| 66 | Nhu NT ^66^  Vietnam | 2013 | Clinical | NR | 7  (9.6) | Children<16 | Prospective  Consecutive | 73  (73) | Sputum (126), gastric fluid (49), CSF (5), pleural fluid (3) | Liquid culture | As for Xpert | Culture or smear |
| 67 | Nhu NT ^67^  Vietnam | 2014 | Clinical | NR | 79  (20.8) | Adult>18 | Prospective  Random | 379  (379) | CSF (379) | Liquid culture | As for Xpert | Clinical algorithm score |
| 68 | Nicol MP ^68^  South Africa | 2011 | Clinical | 250  (55.3) | 108  (23.9) | Children≤15  (19.4 months, 11.1–46.2 months) | Prospective  Consecutive | 452  (452) | Sputum (452) | Liquid culture | As for Xpert | None |
| 69 | Nicol MP ^69^  South Afirca | 2013 | Clinical | NR | 17  (14.8) | Children<15  (31 months, 19-57 months) | Prospective  Consecutive | 115  (115) | Stool (115), sputum (115) | Liquid culture | Sputum | None |
| 70 | Ntinginya EN ^70^  Tanzania | 2012 | Laboratory | 89  (40.6) | NR | 33.6% 5-14, 56.8% 15-55, 9.2%>55 | Cross-sectional  Consecutive | 219  (219) | Sputum  (219) | Solid or liquid culture | As for Xpert | None |
| 71 | Ou X ^71^  China | 2014 | Laboratory | 1,741  (70.9) | NR | NR | Unspecified  Consecutive | 2,454  (2,094) | Sputum (2,454) | Solid culture | As for Xpert | None |
| 72 | O'Grady J ^72^  Zambia | 2012 | Clinical | 446  (50.6) | 595  (67.5) | Adults>15  (35, 28-43) | Prospective  Unspecified | 881  (643) | Sputum  (881) | Liquid culture | As for Xpert | None |
| 73 | Ozkutuk N ^73^  Turkey | 2014 | Laboratory | NR | NR | NR | Unspecified  Unspecified | 2,639  (2,639) | Sputum (721), BAL (757), gastric fluid (94), endotracheal aspirates (30), transtracheal aspirate (9); urine (341), pleural fluid (232), tissue (176), CSF (111), abscesses (94), peritoneal fluid (42), pericardial fluid (18), joint fluid (7), other (7) | Solid and liquid culture | As for Xpert | None |
| 74 | Pandie S ^74^  South Africa | 2014 | Clinical | 93  (62) | 105  (74) | Adult≥18  (34, 29-42) | Prospective  Consecutive | 151  (151) | Pericardial fluid (151) | Liquid culture | As for Xpert | Culture and/or histology |
| 75 | Pang Y ^75^  China | 2014 | Clinical | 128 | NR | Children<14 | Prospective  Consecutive | 211  (211) | Gastric lavage aspirates (211) | Liquid culture | As for Xpert | Smear or culture positive, and/or positive pathological examination, and/or effect to anti-TB regimen, and/or clinical symptoms or chest radiological features |
| 76 | Park KS ^76^  South Korea | 2013 | Clinical | NR | NR | NR | Prospective  Consecutive | 320  (320) | Respiratory specimens (320) | Solid and liquid culture | As for Xpert | None |
| 77 | Patel VB ^77^  South Africa | 2013 | Clinical | 54  (38.6) | 125  (89.3) | Median 33.0, 23.51-42.49 | Prospective  Consecutive | 140  (140) | CSF (140) | Liquid culture | As for Xpert | Definite (culture  or Amplicor PCR) or Probable (anti-TBM treatment initiated but microbiological confirmation lacking), or Non-TBM |
| 78 | Patel VB ^78^  South Africa | 2014 | Clinical | 32  (38.1) | 75  (89.3) | Median 33.0 | Prospective  Consecutive | 84  (144) | CSF (144) | Liquid culture | As for Xpert | Culture and smear microscopy |
| 79 | Peter J ^79^ South Africa Tanzania Zambia | 2015 | Clinical | 311  (53.3) | 564  (96.7) | Adults≥18  (36, 30-41) | Cross-sectional Random | 583  (288) | Sputum (583) | Liquid culture | As for Xpert | None |
| 80 | Porcel JM ^80^  Spain | 2013 | Clinical | 39  (58.2) | 0  (0) | Median 50.3 | Prospective  Consecutive | 67  (67) | Pleural fluid specimens (67) | Solid culture | As for Xpert | None |
| 81 | Peter JG ^81^  South Africa | 2012 | Clinical | NR | 113  (100) | Median 35, IQR 28-38 | Prospective  Random | 113  (175) | Urine  (175) | Liquid culture | Sputum, extra-pulmonary samples | None |
| 82 | Pinyopornpanish K ^82^  Thailand | 2015 | Clinical | 34  (59.6) | 15  (26.3) | ≥15  (55.6, 35.5-75.7) | Cross-sectional  Consecutive | 57  (109) | Sputum (109) | Liquid culture | As for Xpert | None |
| 83 | Rachow A ^83^  Tanzania | 2011 | Clinical | 141  (48.3) | 172  (58.9) | Median 39.2 | Unspecified  Consecutive | 292  (172) | Sputum (292) | Solid and liquid culture | As for Xpert | None |
| 84 | Rachow A ^84^  Tanzania | 2012 | Clinical | 85  (51.8) | 93  (56.7) | Children≤14 | Prospective  Consecutive | 164  (164) | Sputum  (164) | Solid and liquid culture | As for Xpert | None |
| 85 | Reither K ^85^  Tanzania  Uganda | 2015 | Clinical | 219  (45.6) | 197  (43.7) | Children<16  (5.6, 2.0-9.8) | Prospective  Consecutive | 451  (451) | Sputum (451) | Solid and liquid culture | As for Xpert | None |
| 86 | Safianowska A ^86^  Poland | 2012 | Laboratory | NR | NR | NR | Retrospective  Unspecified | 213  (213) | NR | Solid culture | As for Xpert | None |
| 87 | Sekadde MP ^87^  Uganda | 2013 | Clinical | 134  (53.6) | 104  (41.6) | Children<12  (36 months, 16-74.5 months) | Cross-sectional  Consecutive | 250  (235) | Sputum  (235) | Solid and liquid culture | As for Xpert | None |
| 88 | Scott LE ^88^  South Africa | 2014 | Laboratory | NR | NR | NR | Unspecified  Unspecified | 7,916  (1,042) | Cerebrospinal fluid (2,719), Fine-needle aspirate (2,536), Fluid (pleural, ascitic, other) (2,008), Pus (417), Tissue biopsy (184), Dialysis fluid or urine (35), Scrapings (7), Bone (5), Stool (3), Catheter tip (2) | Liquid culture | As for Xpert | None |
| 89 | Shah M ^89^  Uganda | 2014 | Clinical | 74  (35.6) | 208  (100) | Median 33.0, IQR 26.5-38.0 | Prospective  Consecutive | 208  (208) | Sputum (208) | Culture | As for Xpert | None |
| 90 | Sohn H ^90^  Canada | 2014 | Clinical | 279  (55.6) | 12  (2.7) | Adult≥18  (44, 31-61) | Unspecified  Consecutive | 502  (501) | Sputum (502) | Culture | As for Xpert | None |
| 91 | Solomons RS ^91^  South Africa | 2015 | Clinical | 56  (55.4) | 8  (7.9) | Median 35.1 months, IQR 19.2-55.0 months | Prospective  Random | 101  (101) | CSF (101) | Liquid culture | As for Xpert | Definite (AFB and/or culture) or Probable or Possible (based on a scoring system) |
| 92 | Ssengooba W ^92^  Uganda | 2014 | Clinical | 155  (36.6) | 424  (100) | Median 32, IQR 32-34 | Prospective  Unspecified | 424  (424) | Sputum (424) | Liquid culture | As for Xpert | None |
| 93 | Teo J ^93^  Singapore | 2011 | Laboratory | NR | NR | NR | Unspecified  Unspecified | 162  (153) | Sputum (124), BAL (5), TA (2), gastric aspirates (5), urine (3), body fluid (13), miscellaneous samples (10) | Solid and liquid culture | As for Xpert | None |
| 94 | Theron G ^94^  South Africa | 2011 | Clinical | 325  (67.7) | 130  (27.1) | Adults≥18  (36, 18-83) | Unspecified  Consecutive | 480  (480) | Sputum  (480) | Liquid culture | As for Xpert | None |
| 95 | Theron G ^95^  South Africa | 2013 | Clinical | 83  (54) | 46  (29.9) | Adult≥18  (46.1, 33.1-55.7) | Prospective  Consecutive | 154  (152) | BAL (154) | Liquid culture | As for Xpert | Culture and/or histology |
| 96 | Theron G ^96^  South Africa  Zimbabwe  Zambia  Tanzania | 2014 | Clinical | 859  (57.2) | 895  (59.6) | Median 37, IQR 30-46 | Unspecified  Random | 1,502  (729) | Sputum (1,502) | Culture | As for Xpert | None |
| 97 | Trajman A ^97^  Brazil | 2014 | Clinical | 74  (79.6) | 5  (5.4) | Median 50, IQR 40-57 | Unspecified  Unspecified | 93  (59) | Pleural fluid (93) | Liquid culture | As for Xpert | Bacteriology (culture and/or smear) or histology |
| 98 | Tortoli E ^98^  Italia | 2012 | Laboratory | NR | NR | NR | Retrospective  Consecutive | 1474  (1474) | Biopsy specimens (368), pleural fluid (330), gastric aspirate (224), pus (195), CSF (133), urine (130), cavitary fluid (94) | Solid and fluid culture | As for Xpert | None |
| 99 | Vadwai V ^99^  India | 2011 | Clinical | 251  (45.9) | 16  (2.9) | Median 37, IQR 8 months-94 | Unspecified  Consecutive | 547  (432) | Biopsy (284), pus (147), body fluids (93), CSF (23) | Solid and liquid culture | As for Xpert | None |
| 100 | van Kampen SC ^100^  Indonesia | 2015 | Clinical | 872  (60.5) | 35  (2.4) | 0.5%<15, 97.7%≥16, 1.8% missing | Unspecified  Consecutive | 1,442  (654) | Sputum (1,442) | Solid and/or liquid culture | As for Xpert | None |
| 101 | Van Rie A ^101^  South Africa | 2013 | Clinical | 170  (49.4) | 344  (100) | Adult≥18  (35.8, 18-73) | Prospective  Consecutive | 344  (344) | FNA specimens (344) | Liquid culture | As for Xpert | Culture and/or microscopy and/or histology |
| 102 | Williamson DA ^102^  New Zealand | 2012 | Clinical | NR | NR | NR | Unspecified  Unspecified | 169  (169) | Respiratory specimens (89); extra-pulmonary specimens (9), MGIT liquid culture vials (71) | Solid and liquid culture | As for Xpert | None |
| 103 | Yin QQ ^103^  China | 2014 | Clinical | 141  (55.3) | NR | Children≤18  (6.1, 0.3-15.3) | Unspecified  Unspecified | 255  (251) | BAL (251) | Liquid culture | As for Xpert | Chinese CCRS (composite clinical reference standard) |
| 104 | Yoon C ^104^  Uganda | 2012 | Clinical | 248  (52.0) | 362  (75.9) | Adults≥18  (33, 27-40) | Prospective  Consecutive | 477  (436) | Sputum  (477) | Solid and liquid culture | As for Xpert | None |
| 105 | Zar HJ ^105^  South Africa | 2012 | Clinical | 294  (55.0) | 117  (21.9) | Children<15  (19.0 months, 11.2-38.3 months) | Unspecified  Consecutive | 535  (396) | Nasopharyngeal specimens, sputum (396) | Liquid culture | As for Xpert | None |
| 106 | Zar HJ ^106^  South Africa | 2014 | Clinical | 181  (47) | 31  (8) | Children<15  (38.3 months, 21.2-56.5 months) | Prospective  Consecutive | 384  (309) | Sputum (309), Nasopharyngeal aspirate specimens (309) | Liquid culture | Sputum | None |

Sample selection: Study units selected prospectively, or retrospectively from existing samples; Consecutive, random or convenience sampling method. ‘Unspecified’ refers to studies where there was no clear indication how the study participants were chosen. solid media culture(Löwensten-Jensen), liquid media culture (Bactec MGIT 960)

**Abbreviations:**

IQR: interquartile range, TA: tracheal aspirate, BA: bronchial aspirate; BAL: bronchoalveolar lavage; LN: lymph node; CSF: cerebrospinal fluid, EPTB: extra-pulmonary tuberculosis; CCRS: composite clinical reference standard; FNA: fine needle aspirate.
