# Supplementary material for: Diagnostic accuracy of Xpert MTB/RIF for tuberculosis detection in different regions with different endemic burden: A systematic review and meta-analysis
Source: PLoS One. 2017 Jul 14;12(7):e0180725. doi: 10.1371/journal.pone.0180725 (PMC5510832; doi:10.1371/journal.pone.0180725)
Supplement: S2 Table — (DOC) [file pone.0180725.s003.doc]

**Diagnostic accuracy of Xpert** **MTB/RIF for tuberculosis detection in different regions with different endemic burden: a systematic review and meta-analysis**

Shiying Li^1^, Bin Liu^1^, Mingli Peng^1^, Min Chen^1^, Wenwei Yin^1^, Hui Tang^1^, Yuxuan Luo^1^, Peng Hu^1*^, and Hong Ren^1*^

1. Key Laboratory of Molecular Biology for Infectious Diseases (Ministry of Education), Institute for Viral Hepatitis, Department of Infectious Diseases, The Second Affiliated Hospital, Chongqing Medical University, Chongqing, PR China.

* Corresponding author at: Hong Ren and Peng Hu, Department of Infectious Diseases, The Second Affiliated Hospital, Chongqing Medical University, 74# Linjiang Road, Chongqing 400010, China. Tel: +86-2363693029, Fax: +86-2363703790. E-mail: renhong0531@vip.sina.com, hp_cq@163.com

**Supplementary table 2. Data of diagnostic accuracy of the study included in the meta-analysis for tuberculosis detection.**

| Study | First author ^Supplementary ref^. | Year | Total samples n (included n) | True positive | False positive | False negative | True negative | Specimen type |
| --- | --- | --- | --- | --- | --- | --- | --- | --- |
| 1 | Abed Al-Darraji HA ^1^ | 2013 | 125 (125) | 8 | 0 | 7 | 110 | Sputum |
| 2 | Ablanedo-Terrazas Y ^2^ | 2014 | 68 (68) | 15 | 0 | 0 | 53 | Lymph node specimens |
| 3 | Al-Ateah SM ^3^ | 2012 | 234 (239) | 59 | 0 | 3 | 177 | Respiratory and non-respiratory specimens |
|  |  |  | smear（+）（42） | 41 | 0 | 0 | 1 |  |
|  |  |  | smear（-）（197） | 18 | 0 | 3 | 176 |  |
|  |  |  | pulmonary（172） | 42 | 0 | 2 | 128 |  |
|  |  |  | extrapulmonary（67） | 17 | 0 | 1 | 49 |  |
| 4 | Alvarez GG ^4^ | 2015 | 344 (413) | 23 | 4 | 4 | 382 | Sputum |
|  |  |  | smear（+）（21） | 19 | 1 | 1 | 0 |  |
|  |  |  | smear（-）（392） | 4 | 3 | 3 | 382 |  |
| 5 | Antonenka U ^5^ | 2013 | 121 (121) | 51 | 2 | 17 | 51 | Respiratory specimens |
|  |  |  | smear（+）（17） | 16 | 0 | 1 | 0 |  |
|  |  |  | smear（-）（80） | 35 | 0 | 16 | 29 |  |
| 6 | Atehortúa S ^6^ | 2015 | 103 (103) | 29 | 6 | 3 | 65 | Respiratory specimens |
| 7 | Bablishvili N ^7^ | 2015 | 366 (366) | 346 | 11 | 0 | 9 | Sputum |
| 8 | Balcells ME ^8^ | 2012 | 160 (160) | 11 | 1 | 1 | 147 | Sputum |
| 9 | Balcha TT ^9^ | 2014 | 812 (135) | 81 | 13 | 41 | 0 | Sputum |
|  |  |  | smear（+）（31） | 27 | 3 | 1 | 0 |  |
|  |  |  | smear（-）（104） | 54 | 10 | 40 | 0 |  |
| 10 | Barmankulova A ^10^ | 2015 | 300 (291) | 191 | 1 | 44 | 55 | Sputum |
| 11 | Barnard M ^11^ | 2012 | 282 (68) | 37 | 0 | 15 | 16 | Sputum |
|  |  |  | smear（+）（22） | 19 | 0 | 2 | 1 |  |
|  |  |  | smear（-）（46） | 18 | 0 | 13 | 15 |  |
| 12 | Bates M ^12^ | 2013 | 94 (94) | 21 | 2 | 5 | 66 | Sputum |
|  |  |  | HIV(+) (62) | 17 | 2 | 4 | 39 |  |
|  |  |  | HIV(-) (22) | 3 | 0 | 0 | 19 |  |
| 13 | Bates M ^13^ | 2013 | 930 (930) | 42 | 7 | 16 | 865 | Sputum, gastric lavage aspirate |
|  |  |  | HIV(+) (279) | 16 | 2 | 6 | 255 |  |
|  |  |  | HIV(-) (594) | 26 | 5 | 9 | 554 |  |
| 14 | Biadglegne F ^14^ | 2014 | 231 (220) | 30 | 62 | 2 | 126 | Lymph node aspirates |
|  |  |  | smear（+）（13） | 12 | 1 | 0 | 0 |  |
|  |  |  | smear（-）（207） | 18 | 61 | 2 | 126 |  |
| 15 | Biadglegne F ^15^ | 2014 | 200 (200) | 11 | 4 | 1 | 184 | Sputum |
| 16 | Blakemore R ^16^ | 2010 | 168 (168) | 79 | 0 | 0 | 89 | Sputum |
| 17 | Boehme CC ^17^ | 2010 | 1730 (1357) | 723 | 12 | 18 | 604 | Sputum |
|  |  | Peru | 341 (313) | 209 | 0 | 2 | 102 |  |
|  |  | Azerbaijan | 353 (219) | 144 | 2 | 5 | 68 |  |
|  |  | South Africa | 726 (601) | 185 | 9 | 8 | 399 |  |
|  |  | India | 310 (224) | 185 | 1 | 3 | 35 |  |
| 18 | Boehme CC ^18^ | 2011 | 6648 (3909) | 933 | 30 | 100 | 2846 | Sputum |
|  |  | Peru | 1185 (1005) | 171 | 3 | 6 | 825 |  |
|  |  | Azerbaijan | 749 (536) | 203 | 4 | 26 | 303 |  |
|  |  | South Africa | 2522 (904) | 201 | 2 | 32 | 669 |  |
|  |  | Uganda | 372 (289) | 121 | 0 | 24 | 144 |  |
|  |  | India | 902 (289) | 101 | 16 | 0 | 671 |  |
|  |  | Philippines | 918 (387) | 136 | 5 | 12 | 234 |  |
|  |  |  | HIV(+) (602) | 173 | 3 | 37 | 389 |  |
|  |  |  | HIV(-)(1395) | 304 | 6 | 31 | 1054 |  |
| 19 | Bowles EC ^19^ | 2011 | 89 (89) | 60 | 2 | 4 | 23 | Sputum, pleural fluid, gastric fluid, bronchial washing |
|  |  |  | smear（+）（41） | 40 | 1 | 0 | 0 |  |
|  |  |  | smear（-）（48） | 20 | 1 | 4 | 23 |  |
| 20 | Carriquiry G ^20^ | 2012 | 131 (131) | 44 | 2 | 1 | 84 | Sputum |
|  |  |  | smear（+）（31） | 31 | 0 | 0 | 0 |  |
|  |  |  | smear（-）（14） | 13 | 0 | 1 | 0 |  |
| 21 | Causse M ^21^ | 2011 | 289 (340) | 39 | 0 | 2 | 299 | Non-respiratory specimens |
| 22 | Chaisson LH ^22^ | 2014 | 139 (142) | 8 | 0 | 1 | 133 | Sputum |
| 23 | Chisti MJ ^23^ | 2014 | 405 (214) | 4 | 17 | 2 | 191 | Sputum |
|  |  |  | smear（+）（1） | 1 | 0 | 0 | 0 |  |
|  |  |  | smear（-）（213） | 3 | 17 | 2 | 191 |  |
| 24 | Ciftçi IH ^24^ | 2011 | 85 (85) | 24 | 1 | 1 | 59 | Sputum, BAL, thorasynthesis fluid, urine |
| 25 | Coetzee L ^25^ | 2014 | 110 (72)  Culture | 21 | 13 | 4 | 34 | FNA |
|  |  |  | CRS | 32 | 2 | 8 | 30 |  |
| 26 | Coleman M ^26^ | 2015 | 50 (50) | 9 | 0 | 4 | 37 | Pleural effusion; Sputum |
| 27 | Darban-Sarokhalil D ^27^ | 2013 | 247 (247) | 101 | 3 | 11 | 132 | Sputum |
| 28 | Deggim V ^28^ | 2013 | 79 (77) | 15 | 2 | 3 | 57 | Respiratory and None-respiratory |
|  |  |  | smear（+）（19） | 14 | 0 | 0 | 5 |  |
|  |  |  | smear（-）（58） | 1 | 2 | 3 | 52 |  |
| 29 | Dorman SE ^29^ | 2012 | 6,893 (6,621) | 117 | 27 | 70 | 6,407 | Sputum |
|  |  |  | smear（+）（87） | 32 | 1 | 1 | 53 |  |
|  |  |  | smear（-）（6,534） | 85 | 26 | 69 | 6,354 |  |
| 30 | Du J ^30^ | 2015 | 126 (126) | 47 | 2 | 8 | 69 | Pleural biopsy specimen |
|  |  |  | 126 (126) | 24 | 1 | 31 | 70 | Pleural fluid specimen |
| 31 | Feasey NA ^31^ | 2013 | 104 (104) | 9 | 0 | 34 | 61 | Blood |
| 32 | Friedrich SO ^32^ | 2014 | 1918 (1918) | 1117 | 394 | 35 | 372 | Sputum |
| 33 | Giang do C ^33^ | 2015 | 150（150） | 26 | 2 | 12 | 110 | Respiratory and non-respiratory specimens |
| 34 | Gu Y ^34^ | 2015 | 60 (60) | 41 | 0 | 9 | 10 | Pus specimens |
| 35 | Hanrahan CF ^35^ | 2014 | 2,406 (2,082) | 299 | 38 | 107 | 1,638 | Sputum |
|  |  |  | smear（+）（210） | 178 | 15 | 8 | 9 |  |
|  |  |  | smear（-）（1,970） | 97 | 56 | 91 | 1,726 |  |
| 36 | Helb D ^36^ | 2010 | 171 (191) | 130 | 0 | 16 | 45 | Sputum |
|  |  | Vietnam | 107 (107) | 67 | 0 | 15 | 25 |  |
|  |  |  | smear（+）（29） | 29 | 0 | 0 | 0 |  |
|  |  |  | smear（-）（78） | 38 | 0 | 15 | 25 |  |
|  |  | Uganda | 64 (84) | 63 | 0 | 1 | 20 |  |
| 37 | Hillemann D ^37^ | 2011 | 521 (477) | 34 | 7 | 10 | 426 | Non-respiratory specimens |
| 38 | Huh HJ ^38^ | 2014 | 300 (303) | 95 | 24 | 9 | 175 | Respiratory specimens |
|  |  |  | smear（+）（100） | 76 | 3 | 3 | 18 |  |
|  |  |  | smear（-）（171） | 19 | 7 | 6 | 139 |  |
| 39 | Hu P ^39^ | 2014 | 1,352 (728) | 291 | 1 | 17 | 419 | Sputum |
| 40 | Iram S ^40^ | 2015 | 245 (245) | 102 | 9 | 0 | 134 | Respiratory and non-respiratory specimens |
|  |  |  | PTB (205) | 98 | 4 | 0 | 103 |  |
|  |  |  | EPTB (40) | 4 | 5 | 0 | 31 |  |
|  |  |  | Pus (19) | 4 | 5 | 0 | 10 |  |
|  | PTB |  | smear（+）（82） | 82 | 0 | 0 | 0 |  |
|  |  |  | smear（-）（123） | 16 | 4 | 0 | 103 |  |
| 41 | Ismail NA ^41^ | 2015 | 404 (404) | 86 | 14 | 5 | 299 | Sputum |
| 42 | Jafari C ^42^  Culture | 2013 | 96 (96) | 6 | 4 | 4 | 82 | Sputum and BAL |
|  | CRS |  | 96 (96) | 8 | 2 | 11 | 75 |  |
| 43 | Khalil KF ^43^ | 2015 | 93 (93) | 78 | 3 | 7 | 5 | BAL |
| 44 | Kim CH ^44^ | 2014 | 171 (171) | 31 | 3 | 5 | 132 | Pulmonary and Non-pulmonary specimens |
|  |  |  | PTB (43) | 31 | 1 | 5 | 6 |  |
|  |  |  | EPTB (6) | 0 | 0 | 0 | 6 |  |
| 45 | Kim CH ^45^ | 2015 | 383 (373) | 48 | 3 | 4 | 318 | Respiratory and non-respiratory specimens |
| 46 | Kim MJ ^46^ | 2015 | 52 (52) | 34 | 1 | 11 | 6 | Respiratory and non-respiratory specimens |
| 47 | Kim SY ^47^ | 2012 | 71 (69) | 43 | 26 | 0 | 0 | Sputum |
|  |  |  | smear（+）（30） | 28 | 2 | 0 | 0 |  |
|  |  |  | smear（-）（39） | 15 | 24 | 0 | 0 |  |
| 48 | Kim YW ^48^ | 2015  (culture) | 1429 (1,540) | 42 | 28 | 20 | 1,450 | Non-respiratory specimens |
|  |  |  | Adults (1,433) | 38 | 26 | 19 | 1,350 |  |
|  |  |  | Children (107) | 4 | 2 | 1 | 100 |  |
|  |  | CRS | 1429 (1,540) | 70 | 0 | 72 | 1,398 | Non-respiratory specimens |
|  |  |  | Adults (1,433) | 64 | 0 | 70 | 1,299 |  |
|  |  |  | Children (107) | 6 | 0 | 2 | 99 |  |
| 49 | Kokuto H ^49^ | 2015 | 93 (93) | 48 | 0 | 8 | 37 | Fecal specimens; sputum |
|  |  |  | smear（+）27 | 27 | 0 | 0 | 0 |  |
|  |  |  | smear（+-）21 | 17 | 0 | 4 | 0 |  |
|  |  |  | smear（-）8 | 4 | 0 | 4 | 0 |  |
| 50 | Kurbatova EV ^50^ | 2013 | 201 (236) | 103 | 16 | 6 | 111 | Sputum |
| 51 | Kwak N ^51^ | 2013 | 681 (661) | 124 | 0 | 32 | 505 | Sputum |
|  |  |  | smear（+）（79） | 56 | 0 | 7 | 16 |  |
|  |  |  | smear（-）（582） | 68 | 0 | 25 | 489 |  |
| 52 | LaCourse SM ^52^ | 2014 | 300 (300) | 2 | 1 | 0 | 297 | Sputum |
|  |  |  | smear（+）（1） | 1 | 0 | 0 | 0 |  |
|  |  |  | smear（-）（299） | 1 | 1 | 0 | 297 |  |
| 53 | Lawn SD ^53^ | 2011 | 468 (445) | 55 | 3 | 20 | 367 | Sputum |
|  |  |  | smear（+）（61） | 21 | 0 | 0 | 0 |  |
|  |  |  | smear（-）（51） | 34 | 3 | 20 | 367 |  |
| 54 | Lawn SD ^54^ | 2012 | 516 (516) | 49 | 4 | 36 | 427 | Sputum |
| 55 | Lee HY ^55^ | 2013 | 132 (132) | 31 | 4 | 7 | 90 | Bronchoscopy specimens |
| 56 | Le Palud P ^56^ | 2014 | 162 (162) | 16 | 2 | 4 | 140 | BA, BAL, BA/BAL mix |
| 57 | Ligthelm LJ ^57^ | 2011 | 48 (48) | 28 | 3 | 1 | 16 | Fine-needle-aspiration biopsy specimen  s |
|  |  |  | smear（+）（24） | 22 | 1 | 1 | 0 |  |
|  |  |  | smear（-）（8） | 6 | 1 | 0 | 1 |  |
| 58 | Lusiba JK ^58^ | 2014 | 116 (116) | 25 | 1 | 62 | 28 | Pleural fluid; pleural tissue |
|  |  |  | HIV(+) (52) | 15 | 1 | 26 | 10 |  |
|  |  |  | HIV(-) (64) | 10 | 0 | 36 | 18 |  |
| 59 | Malbruny B ^59^ | 2011 | 132 (180) | 29 | 2 | 2 | 147 | Respiratory and non-respiratory specimens |
|  |  |  | smear（+）（17） | 15 | 0 | 0 | 2 |  |
|  |  |  | smear（-）（163） | 14 | 2 | 2 | 145 |  |
|  |  |  | pulmonary（91） | 17 | 0 | 0 | 74 |  |
|  |  |  | extrapulmonary（89） | 12 | 2 | 2 | 73 |  |
| 60 | Marlowe EM ^60^ | 2011 | 216 (216) | 116 | 4 | 14 | 82 | Sputum |
|  |  |  | smear（+）（126） | 85 | 4 | 2 | 35 |  |
|  |  |  | smear（-）（90） | 31 | 0 | 12 | 47 |  |
| 61 | Meldau R ^61^ | 2014 | 88 (88) | 9 | 1 | 31 | 47 | Pleural fluid, Sputum, Pleural biopsy |
| 62 | Miller MB ^62^ | 2011 | 90 (112) | 34 | 4 | 3 | 71 | Respiratory and non-respiratory specimens |
|  |  |  | smear（+）（61） | 28 | 3 | 0 | 30 |  |
|  |  |  | smear（-）（51） | 6 | 1 | 3 | 41 |  |
|  |  |  | pulmonary（89） | 27 | 2 | 2 | 58 |  |
|  |  |  | extrapulmonary（23） | 7 | 2 | 1 | 13 |  |
| 63 | Moure R ^63^ | 2012 | 149 (147) | 63 | 0 | 45 | 39 | Extra-pulmonary specimens |
| 64 | Muñoz L ^64^ | 2011 | 122 (124) | 64 | 0 | 21 | 39 | Respiratory and non-respiratory specimens |
|  |  |  | pulmonary（107） | 61 | 0 | 17 | 29 |  |
|  |  |  | extrapulmonary（17） | 3 | 0 | 4 | 10 |  |
| 65 | Myneedu VP ^65^ | 2014 | 134 (120) | 95 | 11 | 0 | 14 | Sputum |
| 66 | Nhu NT ^66^ | 2013 | 73 (73)  Culture | 29 | 0 | 2 | 42 | Sputum, gastric fluid, CSF, pleural fluid |
|  |  |  | Clinical diagnosis | 29 | 0 | 29 | 15 |  |
| 67 | Nhu NT ^67^ | 2014 | 379 (379) | 108 | 1 | 74 | 196 | CSF |
| 68 | Nicol MP ^68^ | 2011 | 452 (452) | 52 | 6 | 18 | 376 | Sputum |
|  |  |  | HIV(+) (108) | 17 | 0 | 0 | 91 |  |
|  |  |  | HIV(-)(344) | 35 | 6 | 18 | 285 |  |
|  |  |  | smear（+）（27） | 27 | 0 | 0 | 0 |  |
|  |  |  | smear（-）（425） | 25 | 6 | 18 | 376 |  |
| 69 | Nicol MP ^69^ | 2013 | 115 (115) | 8 | 1 | 9 | 97 | Stool |
|  |  |  | HIV(+) (17) | 4 | 0 | 1 | 12 |  |
|  |  |  | HIV(-) (98) | 4 | 1 | 8 | 85 |  |
|  |  |  | 115 (115) | 11 | 3 | 6 | 95 | Sputum |
|  |  |  | HIV(+) (17) | 3 | 1 | 2 | 11 |  |
|  |  |  | HIV(-) (98) | 8 | 1 | 4 | 85 |  |
| 70 | Ntinginya EN ^70^ | 2012 | 219 (219) | 5 | 0 | 0 | 214 | Sputum |
| 71 | Ou X ^71^ | 2014 | 2,454 (2094) | 519 | 152 | 31 | 1,392 | Sputum |
| 72 | O'Grady J ^72^ | 2012 | 881 (643) | 173 | 22 | 28 | 420 | Sputum |
|  |  |  | HIV(+)(408) | 142 | 12 | 19 | 235 |  |
|  |  |  | HIV(-)(196) | 26 | 6 | 9 | 155 |  |
| 73 | Ozkutuk N ^73^ | 2014 | 2,639 (2,639) | 133 | 34 | 47 | 2,425 | Respiratory and non-respiratory specimens |
|  |  |  | smear（+）（133） | 102 | 13 | 0 | 18 |  |
|  |  |  | smear（-）（2,506） | 31 | 21 | 47 | 2,407 |  |
|  |  |  | pulmonary（1,611） | 101 | 18 | 24 | 1,468 |  |
|  |  |  | extrapulmonary（1,028） | 32 | 16 | 23 | 957 |  |
| 74 | Pandie S ^74^ | 2014 | 151 (95) | 44 | 0 | 25 | 26 | Pericardial fluid |
|  |  |  | HIV(+) (60) | 41 | 0 | 14 | 5 |  |
|  |  |  | HIV(-) (27) | 3 | 0 | 11 | 13 |  |
| 75 | Pang Y ^75^ | 2014 | 211 (211)  Culture | 11 | 58 | 6 | 136 | Gastric lavage aspirates |
|  |  |  | CRS | 68 | 1 | 72 | 70 |  |
| 76 | Park KS ^76^ | 2013 | 320 (320) | 19 | 6 | 9 | 286 | Respiratory specimens |
|  |  |  | smear（+）（26） | 10 | 0 | 5 | 11 |  |
|  |  |  | smear（-）（294） | 9 | 6 | 4 | 275 |  |
| 77 | Patel VB ^77^ | 2013 | 140 (140) | 36 | 4 | 22 | 71 | CSF |
|  |  |  | HIV(+) (119) | 36 | 4 | 18 | 61 |  |
|  |  |  | HIV(-) (14) | 0 | 0 | 4 | 10 |  |
| 78 | Patel VB ^78^ | 2014 | 84 (144)culture | 15 | 7 | 16 | 106 | CSF |
|  |  |  | 84 (89)CRS | 18 | 3 | 18 | 50 |  |
| 79 | Peter J ^79^ | 2015 | 583 (288) | 70 | 14 | 22 | 182 | Sputum |
| 80 | Porcel JM ^80^ | 2013 | 67 (67) | 5 | 0 | 28 | 34 | Pleural fluid specimens |
| 81 | Peter JG ^81^ | 2012 | 113 (175) | 54 | 1 | 59 | 61 | Sputum, extra-pulmonary specimens |
| 82 | Pinyopornpanish K ^82^ | 2015 | 57 (109) | 41 | 9 | 2 | 57 | Sputum |
|  |  | 57（106） | HIV(+) 29 | 10 | 0 | 0 | 16 |  |
|  |  |  | HIV(-) 77 | 28 | 2 | 2 | 41 |  |
|  |  | 57（43） | smear（+）(26) | 26 | 0 | 0 | 0 |  |
|  |  |  | smear（-）(17) | 15 | 0 | 2 | 0 |  |
| 83 | Rachow A ^83^ | 2011 | 292 (172) | 61 | 1 | 8 | 102 | Sputum |
|  |  |  | HIV(+) (100) | 44 | 1 | 6 | 49 |  |
|  |  |  | HIV(-)(72) | 17 | 0 | 2 | 53 |  |
|  |  |  | smear（+）（51） | 50 | 0 | 1 | 0 |  |
|  |  |  | smear（-）（121） | 11 | 1 | 7 | 102 |  |
| 84 | Rachow A ^84^ | 2012 | 164 (164) | 21 | 4 | 7 | 132 | Sputum |
|  |  |  | smear（+）（7） | 7 | 0 | 0 | 0 |  |
|  |  |  | smear（-）（157） | 14 | 4 | 7 | 132 |  |
| 85 | Reither K ^85^ | 2015 | 451 (451) | 25 | 0 | 12 | 414 | Sputum |
|  |  |  | HIV(+)(197) | 8 | 0 | 2 | 187 |  |
|  |  |  | HIV(-)(247) | 17 | 0 | 10 | 220 |  |
| 86 | Safianowska A ^86^ | 2012 | 213 (213) | 18 | 1 | 4 | 190 | NR |
|  |  |  | smear（+）（22） | 14 | 1 | 0 | 7 |  |
|  |  |  | smear（-）（189） | 4 | 0 | 4 | 181 |  |
| 87 | Sekadde MP ^87^ | 2013 | 250 (235) | 27 | 7 | 7 | 194 | Sputum |
|  |  |  | HIV(+) (99) | 9 | 4 | 2 | 84 |  |
|  |  |  | HIV(-) (136) | 18 | 3 | 5 | 110 |  |
|  |  |  | smear（+）（15） | 13 | 1 | 1 | 0 |  |
|  |  |  | smear（-）（220） | 14 | 6 | 6 | 194 |  |
| 88 | Scott LE ^88^ | 2014 | 7,916 (1,042) | 160 | 62 | 113 | 707 | Extra-pulmonary specimens |
| 89 | Shah M ^89^ | 2014 | 208 (208) | 78 | 2 | 25 | 103 | Sputum |
|  |  |  | smear（+）（43） | 42 | 0 | 1 | 0 |  |
|  |  |  | smear（-）（60） | 36 | 0 | 24 | 0 |  |
| 90 | Sohn H ^90^ | 2014 | 502 (501) | 11 | 1 | 14 | 475 | Sputum |
|  |  |  | smear（+）（11） | 6 | 0 | 1 | 4 |  |
|  |  |  | smear（-）（425） | 5 | 1 | 13 | 406 |  |
| 91 | Solomons RS ^91^ | 2015 | 101 (101) | 14 | 0 | 41 | 46 | CSF |
| 92 | Ssengooba W ^92^ | 2014 | 424 (424) | 94 | 10 | 29 | 291 | Sputum |
| 93 | Teo J ^93^ | 2011 | 162 (153) | 70 | 8 | 7 | 68 | Respiratory and non-respiratory specimens |
|  |  |  | smear（+）（58） | 47 | 5 | 0 | 6 |  |
|  |  |  | smear（-）（95） | 23 | 3 | 7 | 62 |  |
|  |  |  | pulmonary（122） | 56 | 5 | 6 | 55 |  |
|  |  |  | extrapulmonary（31） | 14 | 3 | 1 | 13 |  |
| 94 | Theron G ^94^ | 2011 | 480 (480) | 111 | 19 | 30 | 320 | Sputum |
|  |  |  | HIV(+) (130) | 32 | 7 | 14 | 77 |  |
|  |  |  | HIV(-)(286) | 68 | 9 | 14 | 195 |  |
|  |  |  | smear（+）（95） | 89 | 0 | 5 | 1 |  |
|  |  |  | smear（-）（385） | 22 | 19 | 25 | 319 |  |
| 95 | Theron G ^95^ | 2013  culture | 154 (152) | 25 | 5 | 2 | 120 | BAL |
|  |  |  | HIV(+) (44) | 6 | 2 | 2 | 34 |  |
|  |  |  | HIV(-) (84) | 12 | 3 | 0 | 69 |  |
|  |  |  | smear（-）(135) | 9 | 4 | 2 | 120 |  |
|  |  | CRS | 154 (152) | 25 | 5 | 3 | 119 |  |
|  |  |  | HIV(+) (44) | 6 | 2 | 2 | 34 |  |
|  |  |  | HIV(-) (84) | 12 | 3 | 0 | 69 |  |
|  |  |  | smear（-）(135) | 9 | 4 | 3 | 119 |  |
| 96 | Theron G ^96^ | 2014 | 1502 (729) | 154 | 27 | 31 | 517 | Sputum |
|  |  | South Africa | 1502 (296) | 43 | 6 | 14 | 233 |  |
|  |  | Zimbabwe | 1502 (198) | 35 | 7 | 5 | 151 |  |
|  |  | Zambia | 1502 (198) | 65 | 11 | 9 | 113 |  |
|  |  | Tanzania | 1502 (37) | 11 | 3 | 3 | 20 |  |
| 97 | Trajman A ^97^ | 2014 | 93 (59) | 1 | 0 | 32 | 26 | Pleural fluid |
| 98 | Tortoli E ^98^ | 2012 | 1474 (1474) | 188 | 32 | 50 | 1204 | Extra-pulmonary specimens |
| 99 | Vadwai V ^99^ | 2011 | 547 (432) | 125 | 5 | 25 | 277 | Non-respiratory specimens |
| 100 | van Kampen SC ^100^ | 2015 | 1,442 (654) | 339 | 210 | 19 | 86 | Sputum |
| 101 | Van Rie A ^101^ | 2013 | 344 (344) culture | 139 | 23 | 10 | 172 | FNA specimens |
|  |  |  | CRS | 160 | 2 | 42 | 140 |  |
| 102 | Williamson DA ^102^ | 2012 | 169  (169) | 141 | 0 | 0 | 28 | Respiratory; extra-pulmonary specimens, positive MGIT liquid culture vials |
|  |  |  | pulmonary（89） | 67 | 0 | 0 | 22 |  |
|  |  |  | extrapulmonary（9） | 9 | 0 | 0 | 0 |  |
|  |  |  | smear（+）（98） | 76 | 0 | 0 | 22 |  |
| 103 | Yin QQ ^103^ | 2014 | 255 (251) culture | 24 | 20 | 0 | 207 | BAL |
|  |  |  | CRS | 44 | 0 | 39 | 168 |  |
|  |  |  | CRS smear（+）（7） | 7 | 0 | 0 | 0 |  |
|  |  |  | smear（-）（244） | 37 | 0 | 39 | 168 |  |
| 104 | Yoon C ^104^ | 2012 | 477 (436) | 187 | 9 | 50 | 190 | Sputum |
|  |  |  | HIV(+) (328) | 144 | 8 | 39 | 137 |  |
|  |  |  | HIV(-) (107) | 43 | 0 | 11 | 53 |  |
| 105 | Zar HJ ^105^ | 2012 | 535 (396) | 45 | 3 | 18 | 330 | Sputum |
|  |  |  | HIV(+) (80) | 7 | 0 | 1 | 72 |  |
|  |  |  | HIV(-)(315) | 38 | 3 | 17 | 257 |  |
|  |  |  | 535 (396) | 41 | 6 | 22 | 327 | Nasopharyngeal specimens |
|  |  |  | HIV(+) (80) | 8 | 2 | 0 | 70 |  |
|  |  |  | HIV(-)(315) | 33 | 4 | 22 | 256 |  |
| 106 | Zar HJ ^106^ | 2014 | 384 (309) | 18 | 4 | 10 | 277 | Sputum Nasopharyngeal aspirate specimens |
