# Supplementary figures and images for: Diagnostic accuracy of Xpert MTB/RIF for tuberculosis detection in different regions with different endemic burden: A systematic review and meta-analysis
Source: PLoS One. 2017 Jul 14;12(7):e0180725. doi: 10.1371/journal.pone.0180725 (PMC5510832; doi:10.1371/journal.pone.0180725)

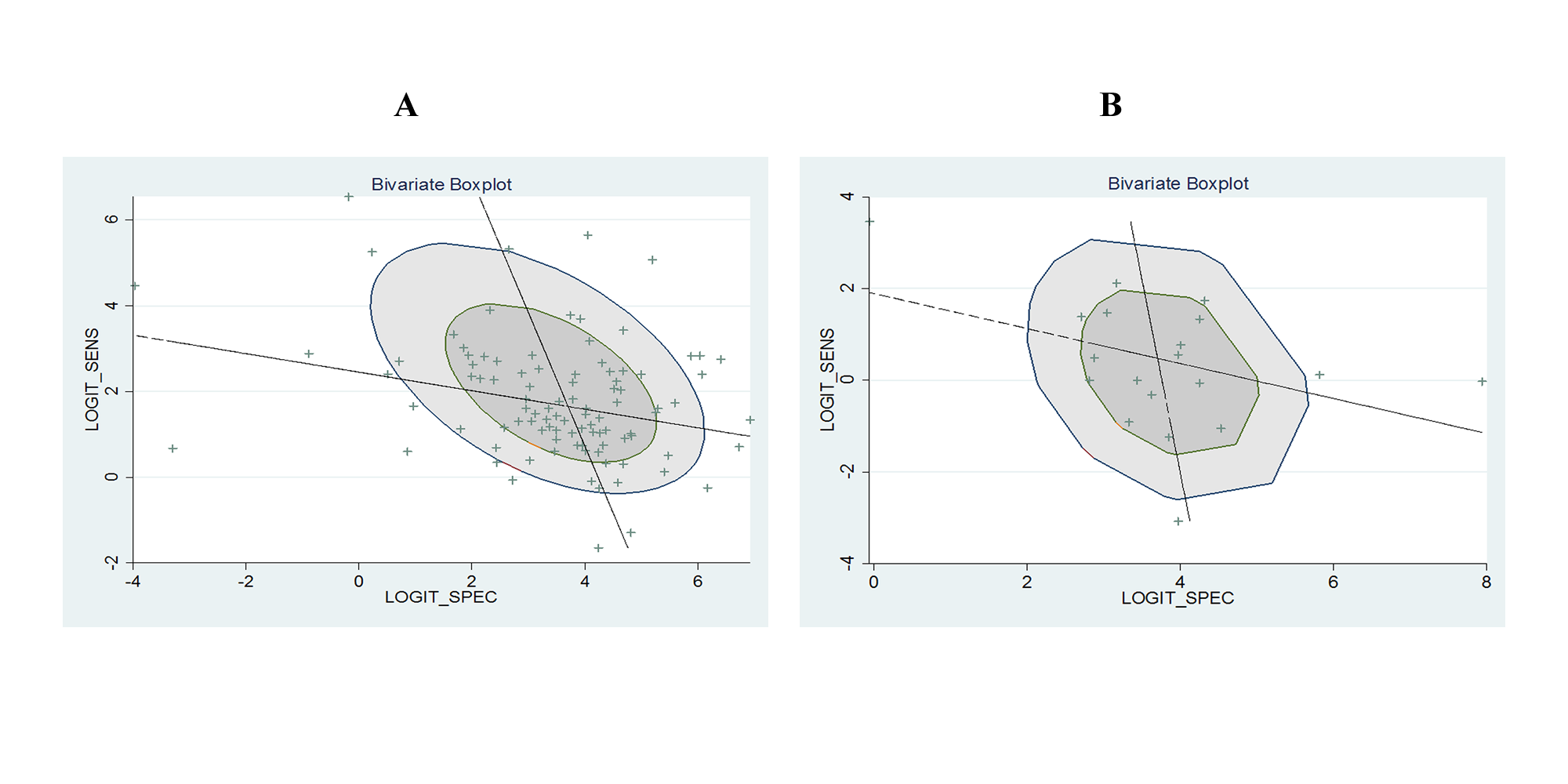

Supplement: S3 Fig — Heterogeneity analyses of included studies for tuberculosis detection versus (A) culture reference standard, (B) composite reference standard. (TIF) [file pone.0180725.s006.tif]

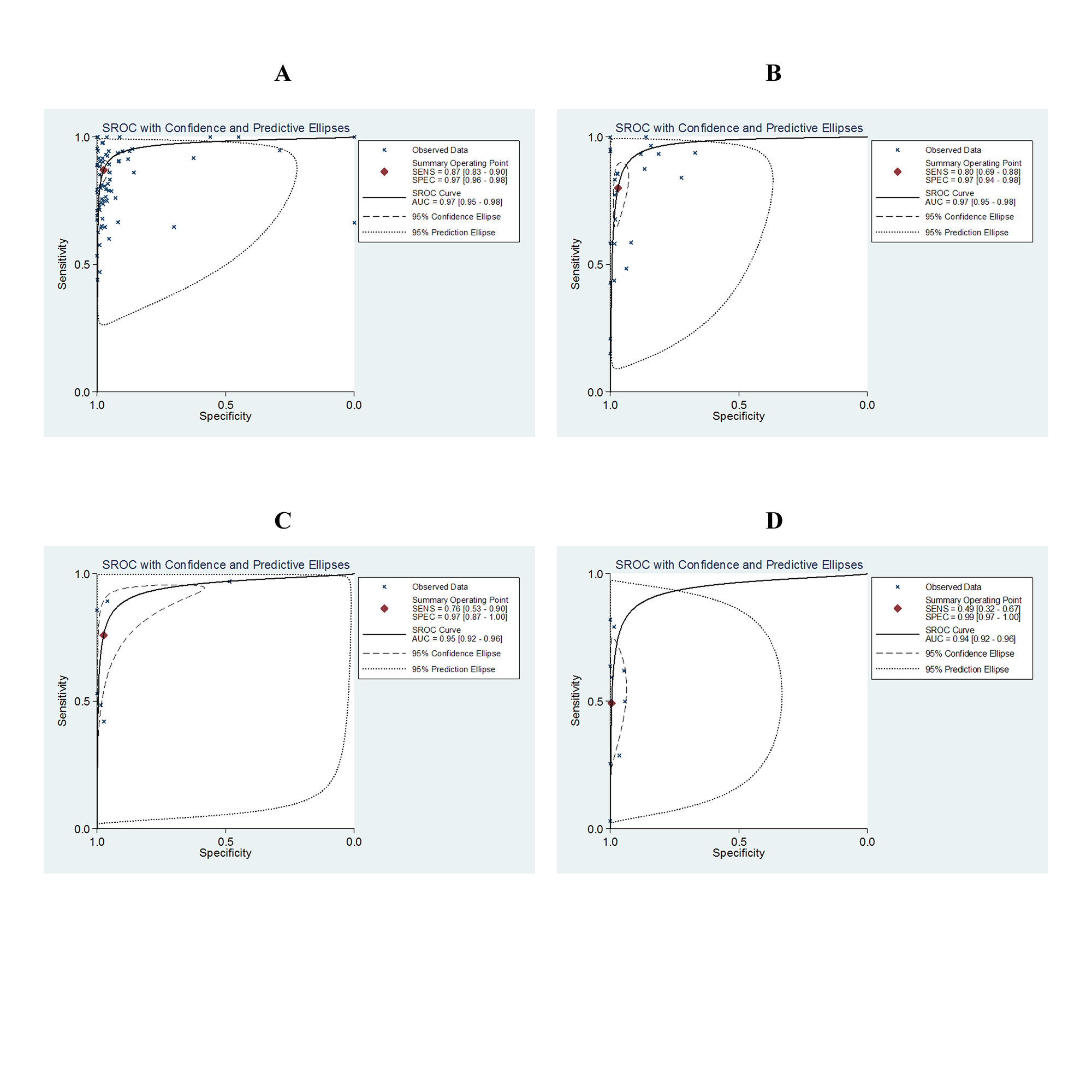

Supplement: S4 Fig — (A) PTB, culture reference standard, (B) EPTB, culture reference standard, (C) PTB, composite reference standard, (D) EPTB, composite reference standard. The point represents the sensitivity and specificity of one study; the summary point represents the summary sensitivity and specificity. (TIF) [file pone.0180725.s007.tif]

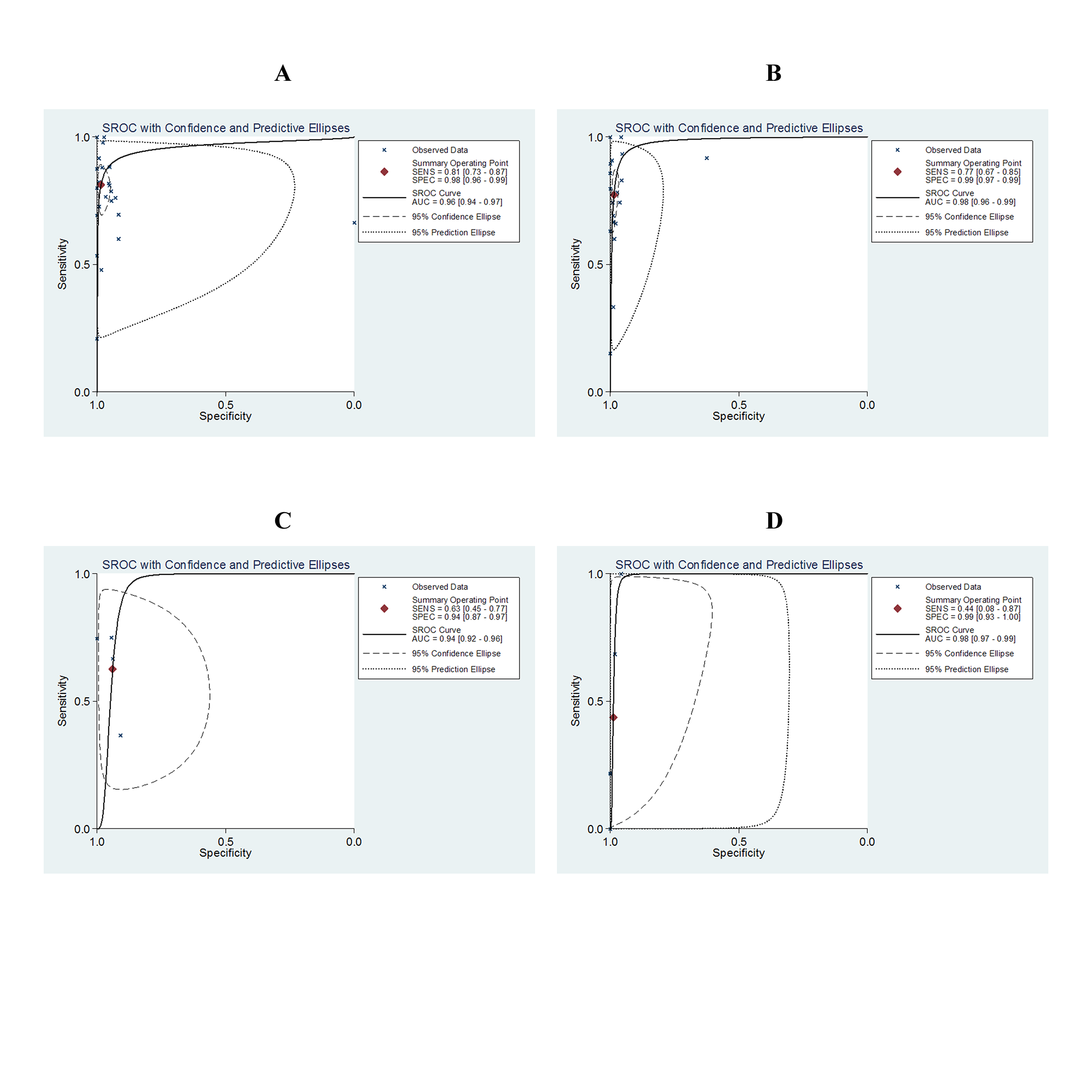

Supplement: S5 Fig — (A) HIV (+), culture reference standard, (B) HIV (-), culture reference standard, (C) HIV (+), composite reference standard, (D) HIV (-), composite reference standard. The point represents the sensitivity and specificity of one study; the summary point represents the summary sensitivity and specificity. (TIF) [file pone.0180725.s008.tif]

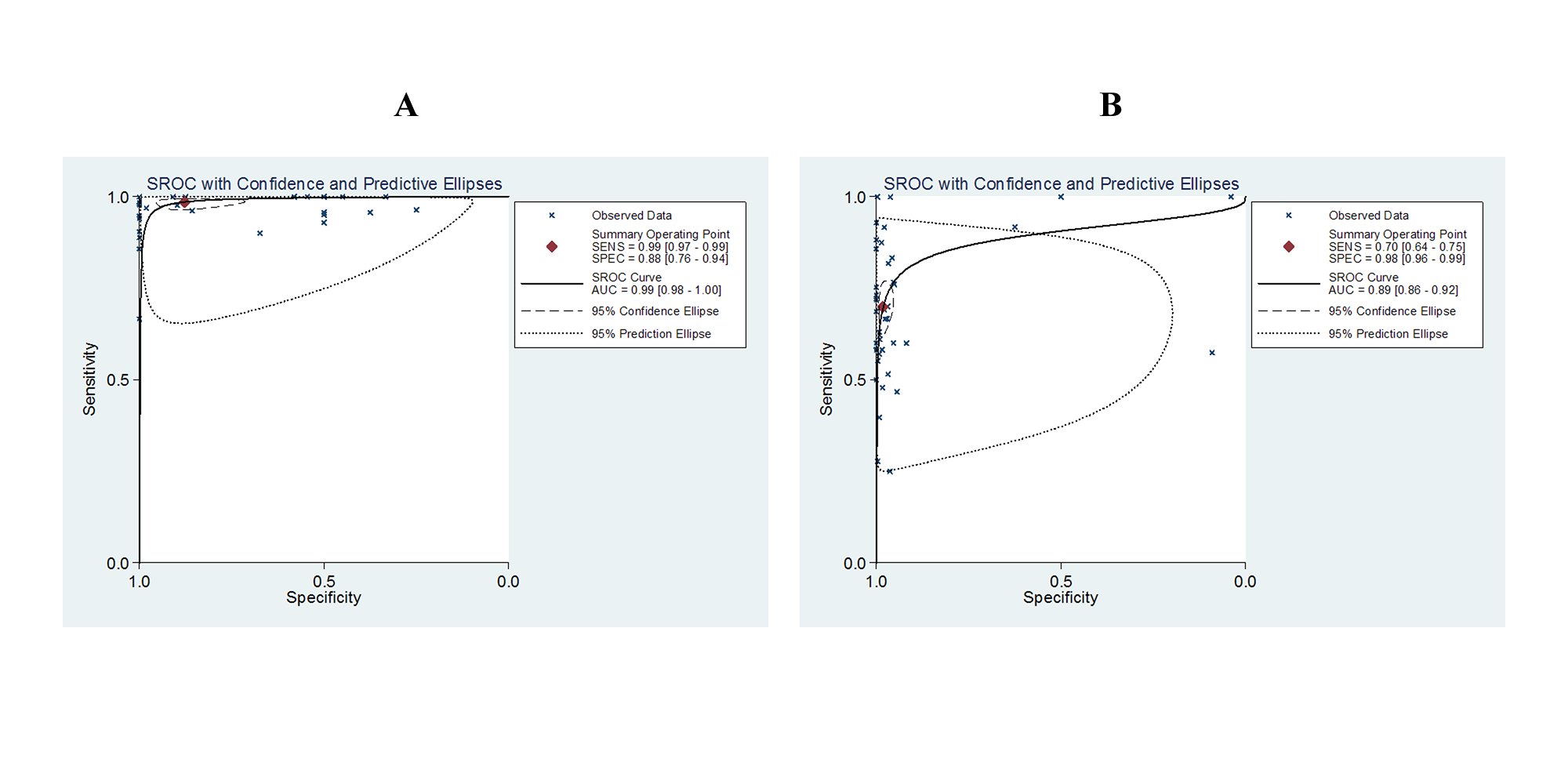

Supplement: S6 Fig — (A) Smear (+), culture reference standard, (B) Smear (-), culture reference standard. The point represents the sensitivity and specificity of one study; the summary point represents the summary sensitivity and specificity. (TIF) [file pone.0180725.s009.tif]

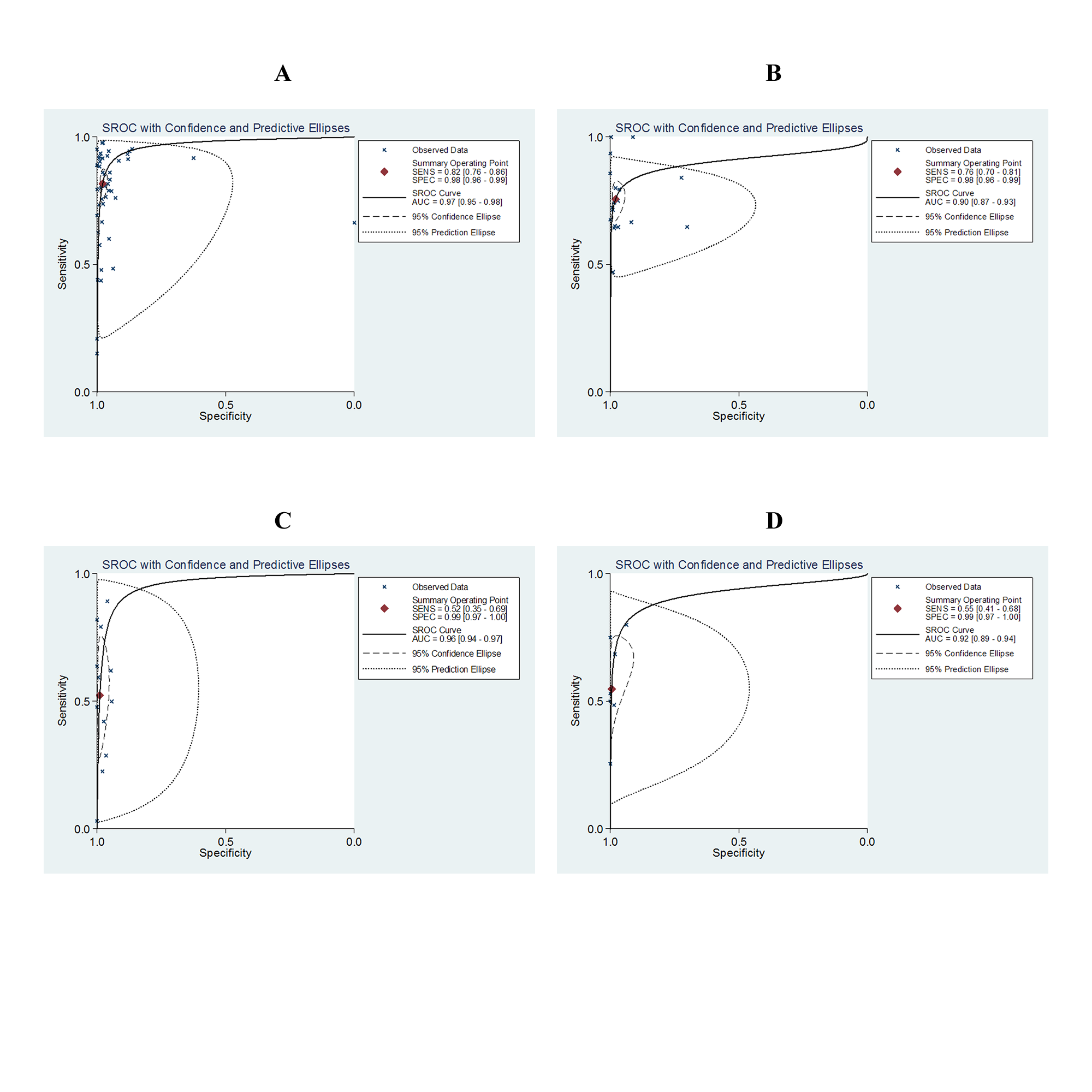

Supplement: S7 Fig — (A) Adults, culture reference standard, (B) Children, culture reference standard, (C) Adults, composite reference standard, (D) Children, composite reference standard. The point represents the sensitivity and specificity of one study; the summary point represents the summary sensitivity and specificity. (TIF) [file pone.0180725.s010.tif]

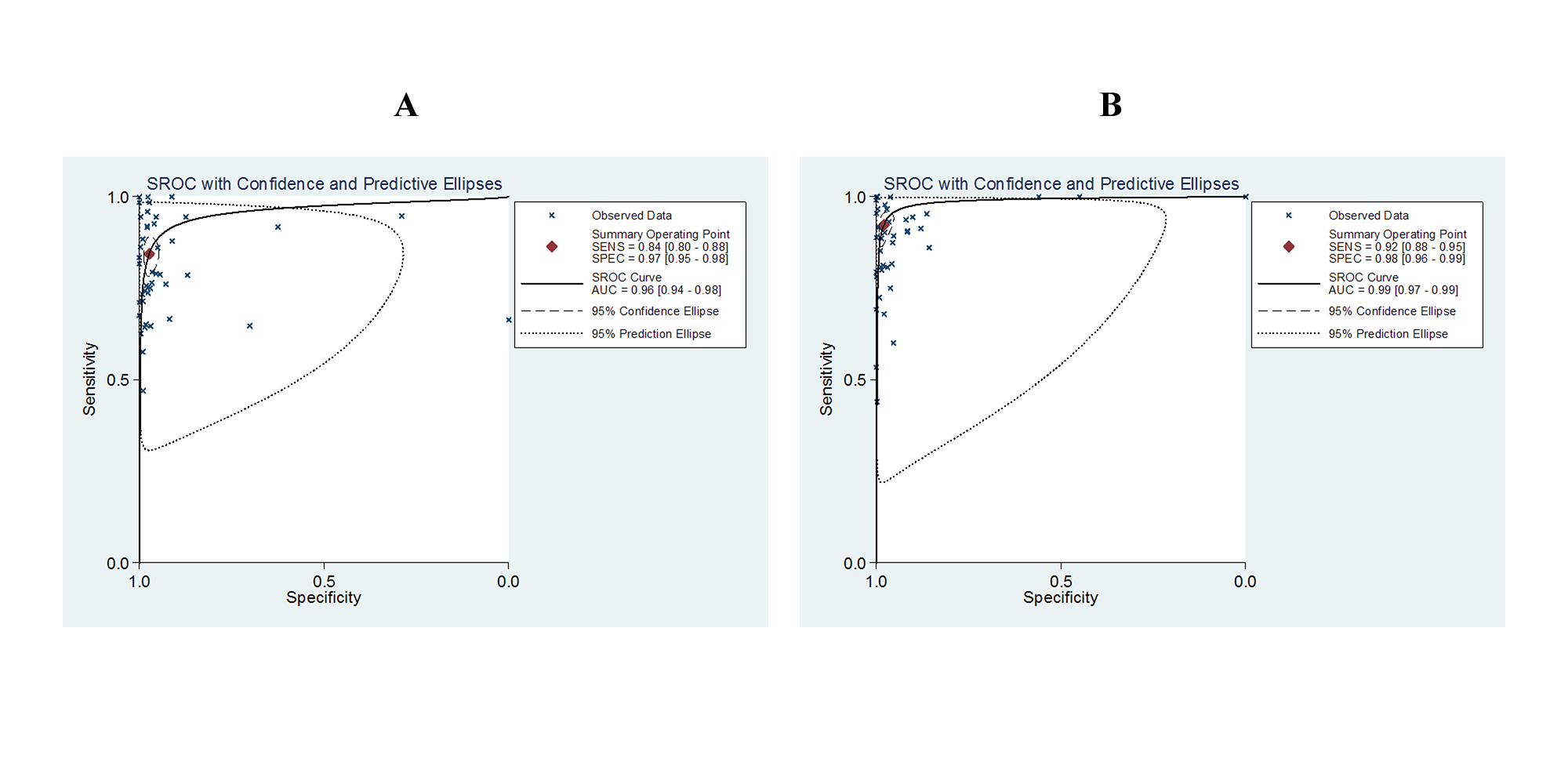

Supplement: S8 Fig — (A) in high TB burden countries, (B) in middle/low TB burden countries. The point represents the sensitivity and specificity of one study; the summary point represents the summary sensitivity and specificity. (TIF) [file pone.0180725.s011.tif]

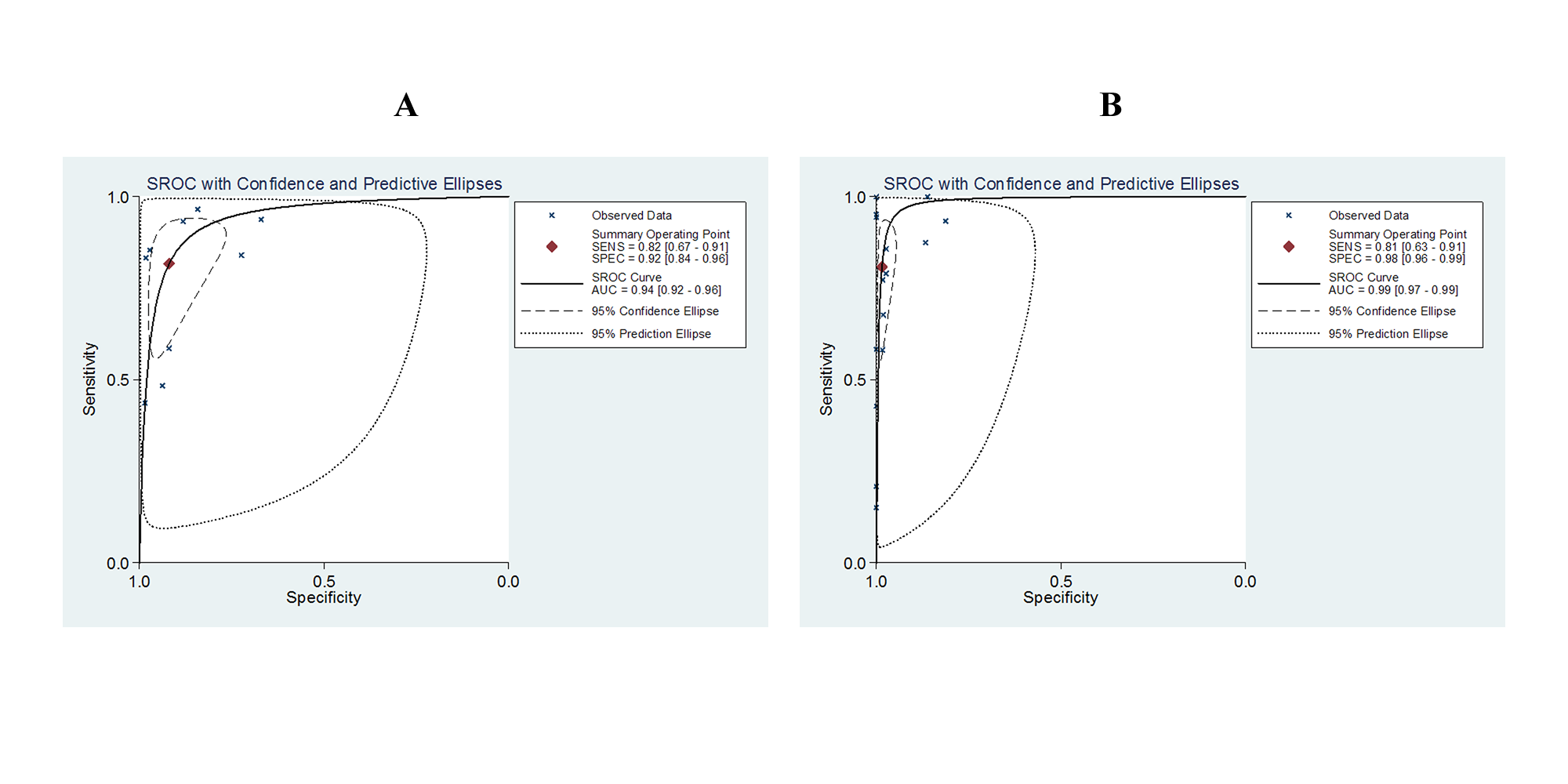

Supplement: S9 Fig — (A) in high TB burden countries, (B) in middle/low TB burden countries. The point represents the sensitivity and specificity of one study; the summary point represents the summary sensitivity and specificity. (TIF) [file pone.0180725.s012.tif]

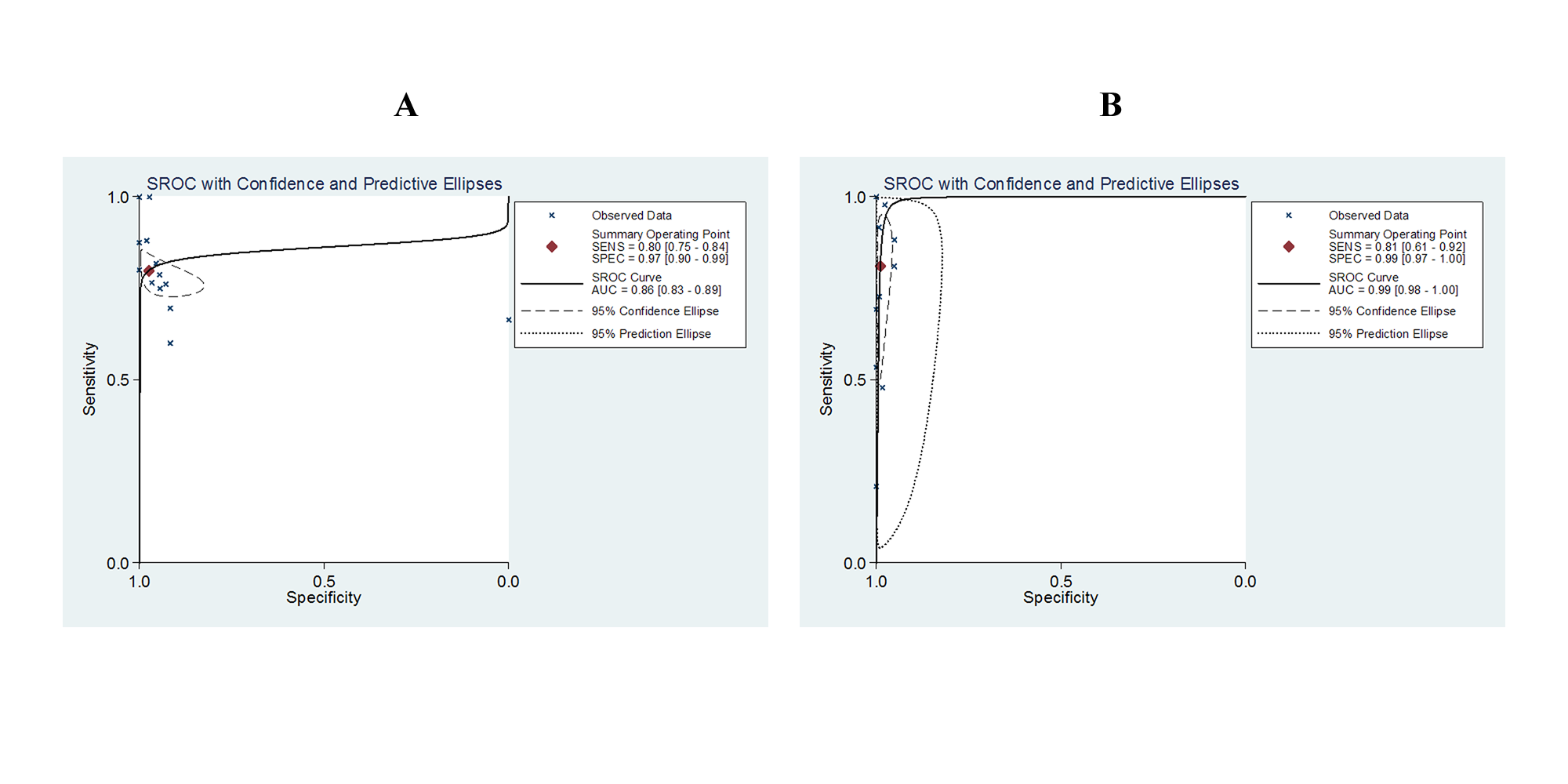

Supplement: S10 Fig — (A) in high TB burden countries, (B) in middle/low TB burden countries. The point represents the sensitivity and specificity of one study; the summary point represents the summary sensitivity and specificity. (TIF) [file pone.0180725.s013.tif]

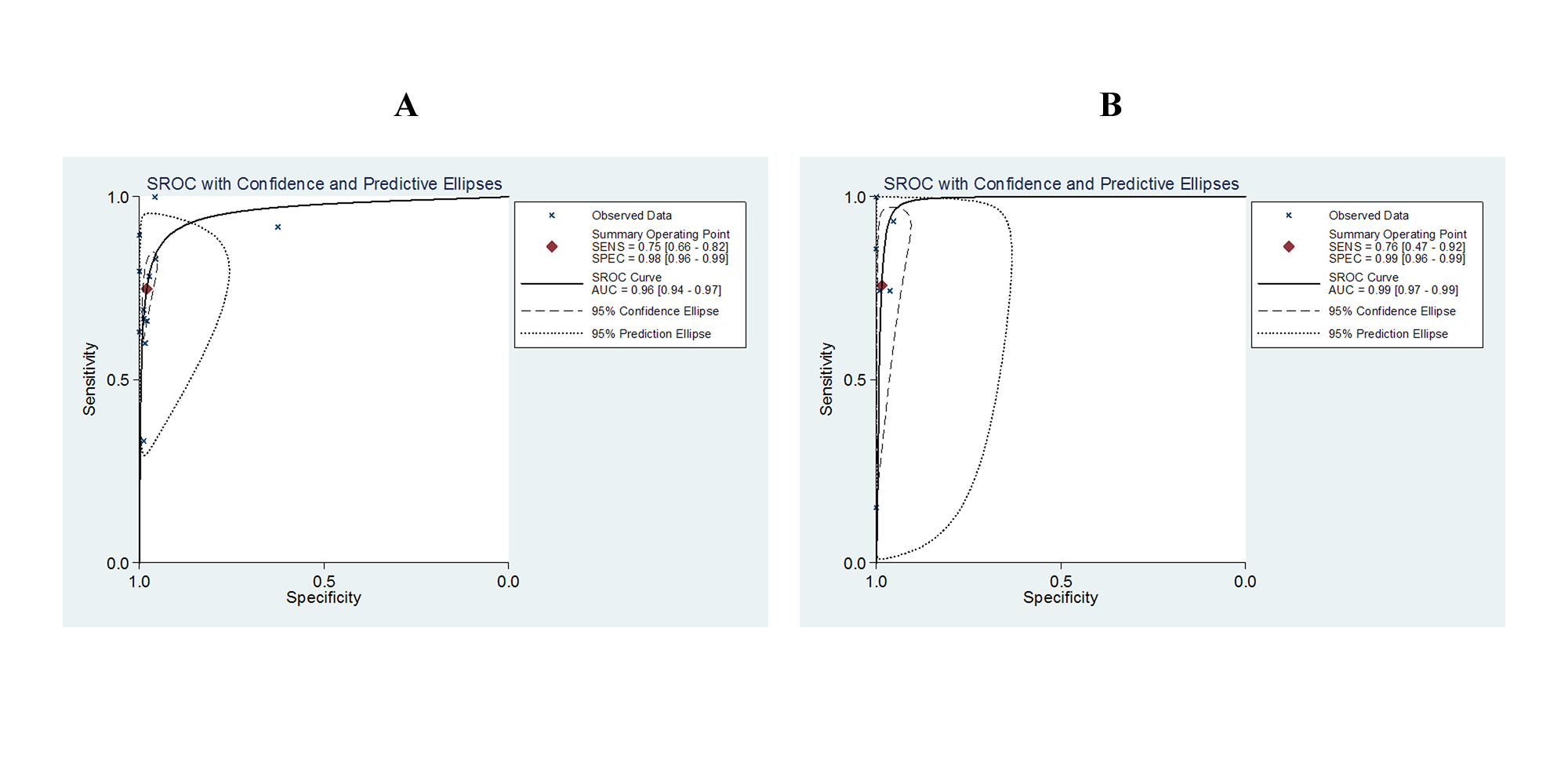

Supplement: S11 Fig — (A) in high TB burden countries, (B) in middle/low TB burden countries. The point represents the sensitivity and specificity of one study; the summary point represents the summary sensitivity and specificity. (TIF) [file pone.0180725.s014.tif]

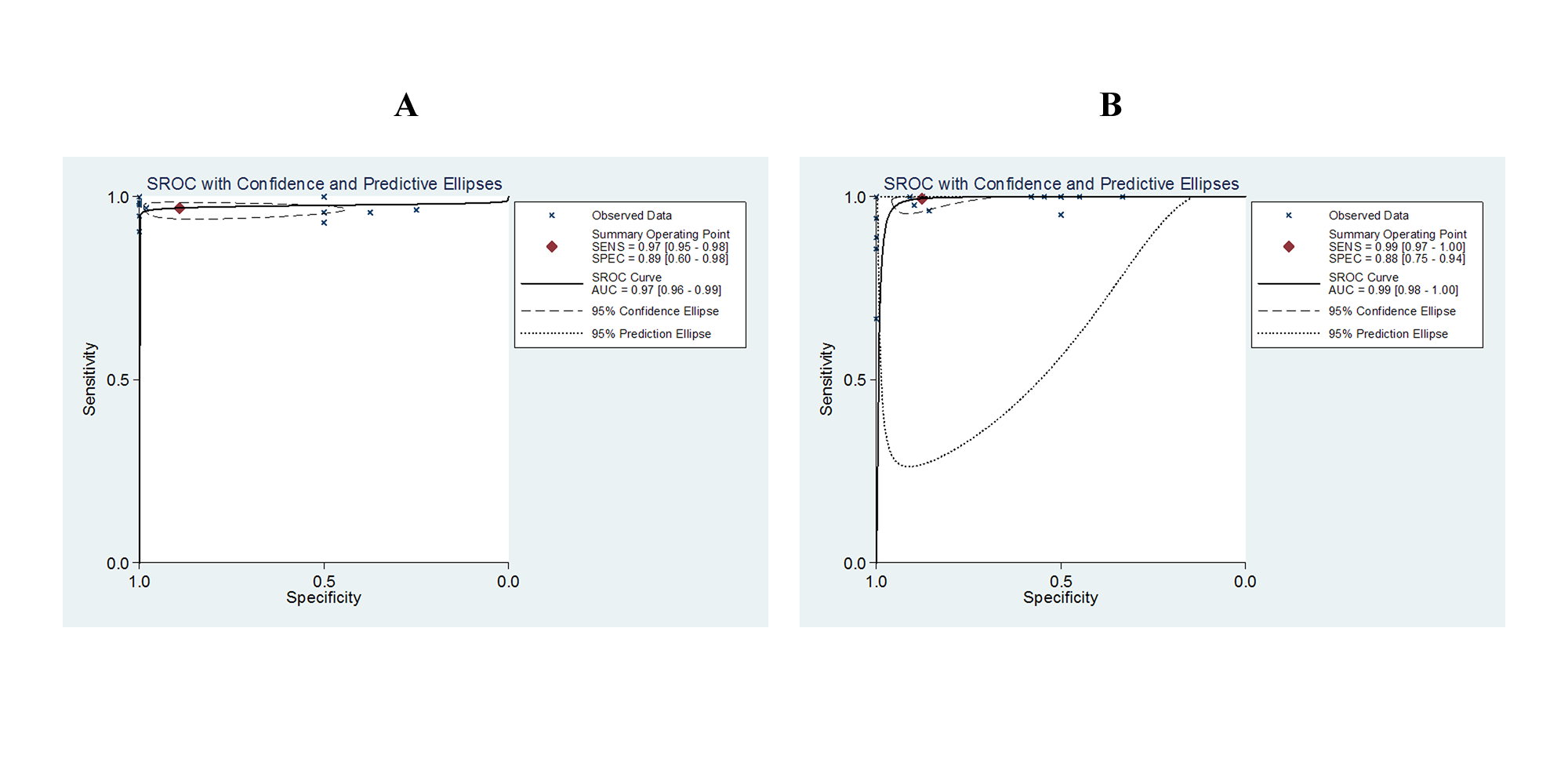

Supplement: S12 Fig — (A) in high TB burden countries, (B) in middle/low TB burden countries. The point represents the sensitivity and specificity of one study; the summary point represents the summary sensitivity and specificity. (TIF) [file pone.0180725.s015.tif]

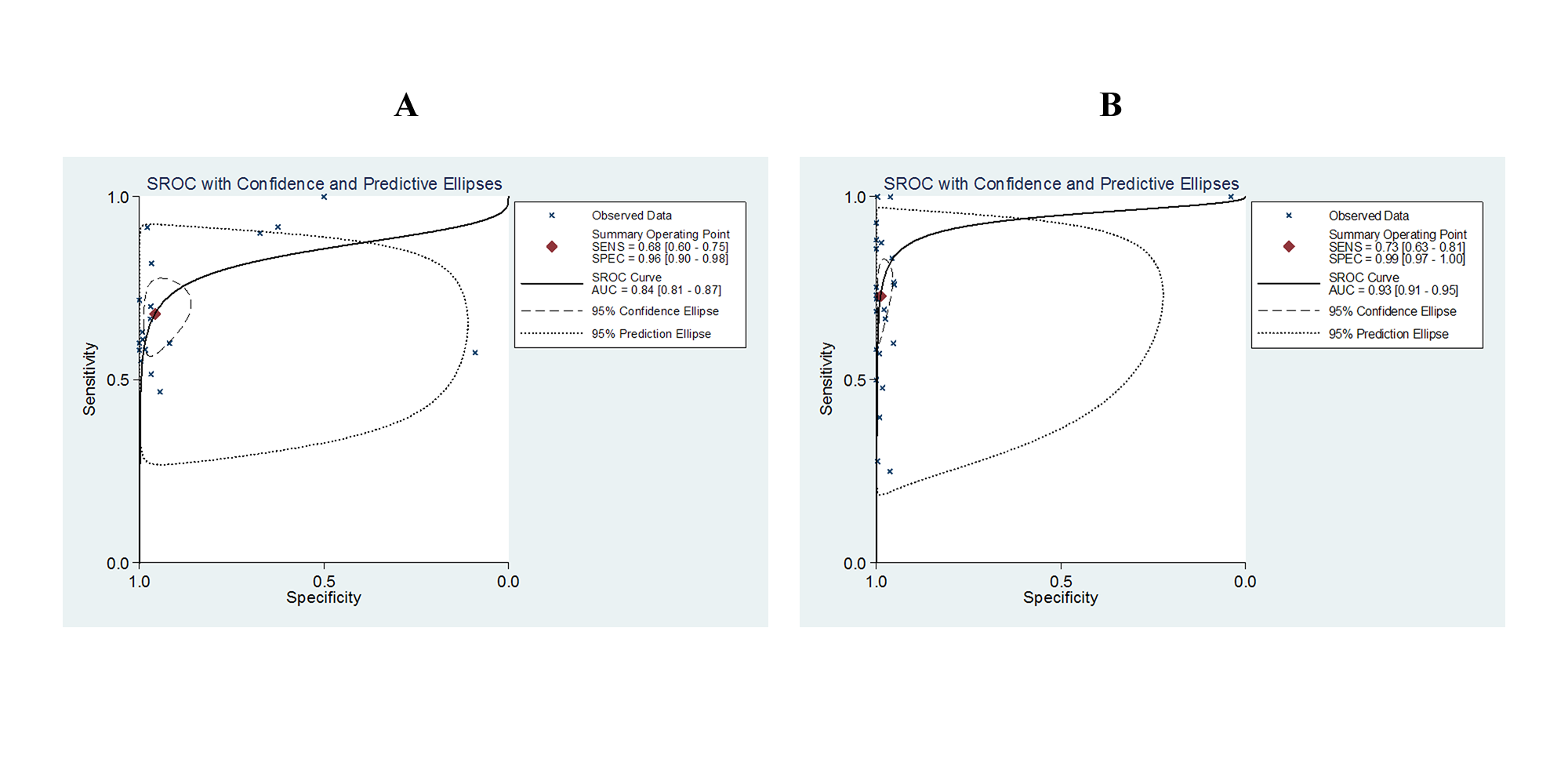

Supplement: S13 Fig — (A) in high TB burden countries, (B) in middle/low TB burden countries. The point represents the sensitivity and specificity of one study; the summary point represents the summary sensitivity and specificity. (TIF) [file pone.0180725.s016.tif]

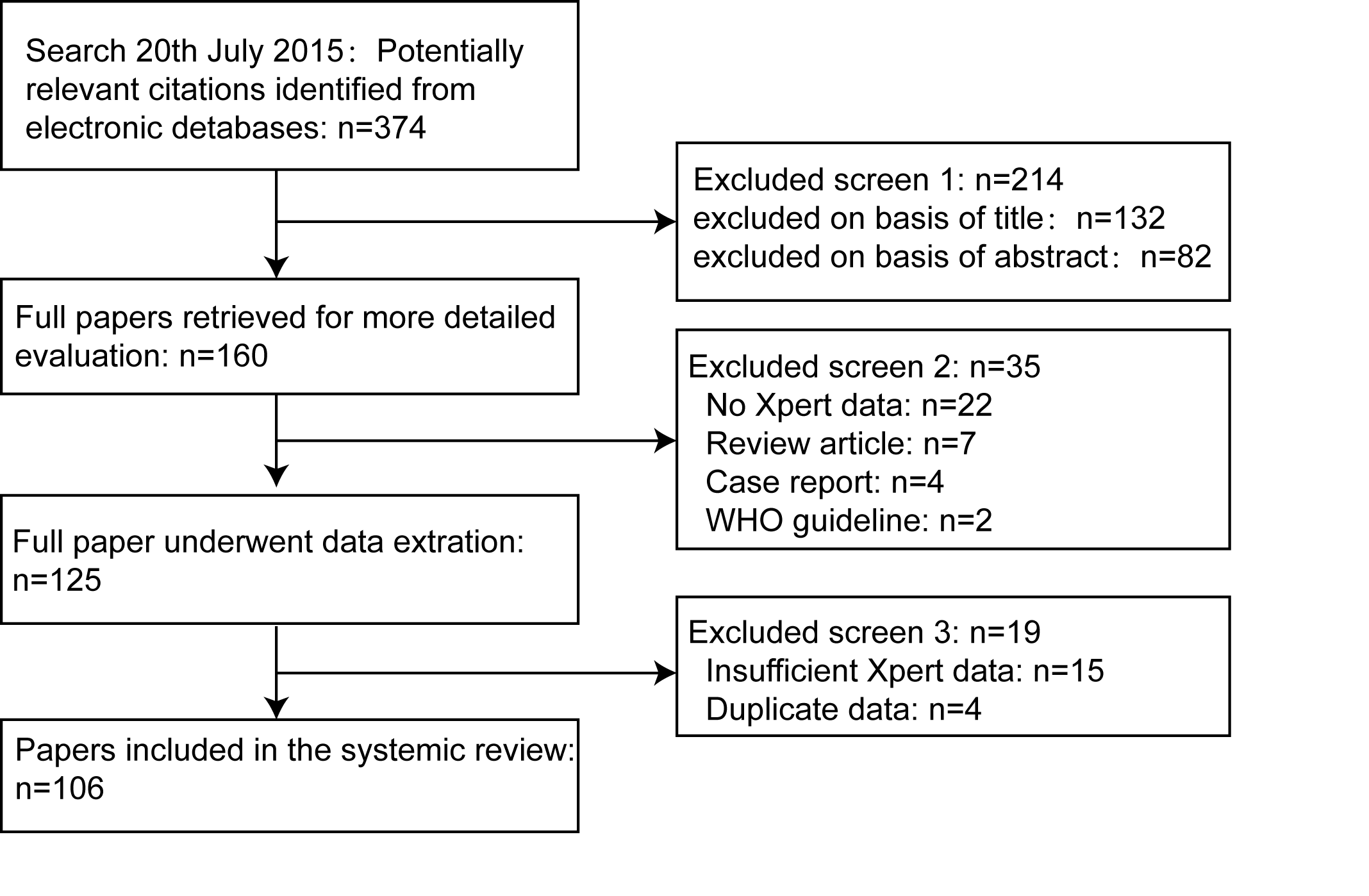

Supplement: S1 Text — (TIF) [file pone.0180725.s017.tif]
